# Allosteric ensembles elucidate mechanisms of inhibition in the human soluble epoxide hydrolase

**DOI:** 10.1038/s42003-026-10100-7

**Published:** 2026-05-05

**Authors:** Qiongju Qiu, Oriol Gracia Carmona, Giancarlo Abis, Franca Fraternali, Maria R. Conte

**Affiliations:** 1 Randall Centre for Cell and Molecular Biophysics, King’s College London, New Hunt’s House, Guy’s Campus, London, UK; 2 Institute of Structural and Molecular Biology, University College London, London, UK

**Keywords:** Biophysical chemistry, Computational biophysics

## Abstract

Human soluble epoxide hydrolase (sEH) is an important modulator of cardiovascular, brain and renal health, and a promising therapeutical target for several pathologies. Its C-terminal domain (CTD) possesses hydrolytic activity which is responsible for decreasing the level of beneficial bioactive epoxy fatty acids. In this work we uncover the mechanisms of allosteric inhibition exerted on sEH CTD through the cysteine residues C522 and C423. In silico investigations coupled with biophysical and biochemical characterizations revealed that allosteric inhibition by the endogenous lipid mediator 15-deoxy-Δ^12,14^-Prostaglandin J_2_ (15d-PGJ_2_) at C423 and C522 operates through distinct structural and dynamic mechanisms. Furthermore, the two allosteric sites C522 and C423 differ in their ability to generate an allosteric response upon binding of different effectors, in that association of divalent copper ions at C423 caused inhibition, whilst binding at C522 did not. The newly generated knowledge of the chemical properties of the effectors, their distal binding site and their specific allosteric signaling to functional sites will be instrumental to develop allostery-based therapeutics for sEH.

## Abstract (web summary)

Combined computational and experimental analysis reveals how electrophilic lipids and Cu²⁺ allosterically inhibit the human soluble epoxide hydrolase via structural rearrangements and inter-residue communication pathways.

## Introduction

Human soluble epoxide hydrolase (sEH) (EC 3.3.2.10; EPHX2) is a dimeric bifunctional enzyme comprising an N-terminal domain (NTD) functioning as a phosphatase, and a C-terminal domain (CTD) with hydrolase activity^1^. sEH participates in fatty acid metabolism downstream the arachidonic acid pathway, where it hydrolyzes beneficial epoxy fatty acids (EpFAs) to their less active 1,2-diols^1,2^. These EpFAs are reported to have cardioprotective, anti-inflammatory and anti-nociceptive effects^2,3^. Stabilization of beneficial EpFA levels through inhibition of sEH CTD has shown antihypertensive, anti-inflammatory and analgesic effects, thus making sEH an attractive therapeutical target for multiple ailments^3–7^.

**Fig. 1:**
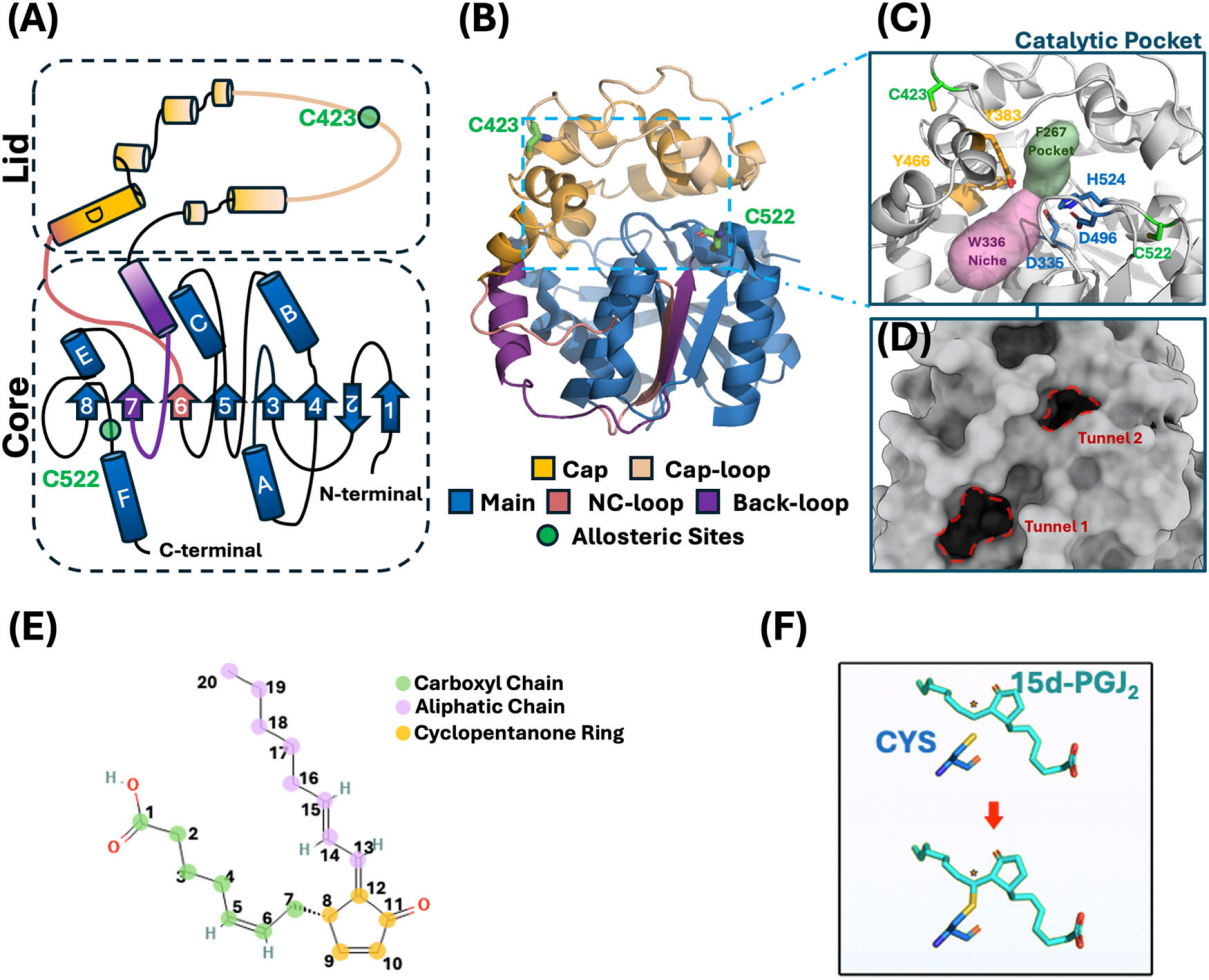
Molecular details of sEH C-terminal domain (CTD) and allosteric inhibitor 15-Deoxy-Δ^12,14^-prostaglandin J_2_ (15d-PGJ_2_). **A** Schematic of sEH CTD secondary structure. sEH CTD adopts the characteristic α/β-hydrolase fold, comprising eight β-strands (β1-β8) and six α-helices (αA-αF). Additional α-helices present in sEH have been left unlabeled for clarity. The main, cap, cap-loop, NC-loop, and back-loop regions are colored in blue, yellow, wheat, pink, and purple, respectively. The allosteric sites C423 and C522 are marked by green dots. **B** Crystal structure of sEH CTD wildtype (WT) (PDB ID: 6I5E, chain A) with all regions and allosteric sites colored as in (**A**). **C** Close-up of the catalytic site showing epoxide positioners (Y383 and Y466) and catalytic triad (D335, D496, H524) in sticks, and the W336 niche and F267 pocket in pink and green surface, respectively. **D** Surface view of sEH CTD catalytic pocket, with Tunnel 1 and Tunnel 2 highlighted by red dashed lines. The orientation is the same as in (**C**). **E** Chemical structure of 15d-PGJ_2_. Its carboxyl chain (C1–C7), aliphatic chain (C13–C20), and cyclopentanone ring (C8–C12) are colored with green, pink, and yellow circles, respectively. **F** Schematic representation of a Michael addition reaction between the 15d-PGJ_2_ electrophilic carbon and the nucleophilic thiol of cysteine (CYS).

Structurally, sEH CTD belongs to the superfamily of α/β-hydrolases, characterized by eight β-strands arranged in a central β-sheet and flanked by six α-helices^8,9^ (Fig. 1A, B). Five sub-regions, termed main (encompassing residues 230–348 and 494–545), cap (370–409 and 444–468), cap-loop (410–443), NC-loop (349–369), and back-loop (240–264)^10^ have been structurally delineated in epoxide hydrolases, and further grouped into two distinct parts of the protein, specifically the core region (formed by the main, NC-loop, and back-loop) and the lid region (containing the cap and cap-loop)^10,11^ (Fig. 1A, B). Notably, sEH CTD contains six extra α-helices additional to the common epoxide hydrolase fold, specifically one located in the back-loop and five on the cap-loop (left unnumbered in Fig. 1A). The active site of sEH CTD is an L-shaped cavity lodged in between the core and lid regions, featuring at its elbow, called vertex, the catalytic triad (D335, D496 and H524) and the epoxide positioners (Y383, Y466), which mediate epoxy ring hydrolysis and substrate alignment respectively^1^ (Fig. 1C). Two large hydrophobic areas, namely the F267 pocket (encompassing residues F267, L408, L417, M419, V498, W525) and the W336 niche (W336, M339, M469, L499), surround the catalytic triad at either side of the vertex and participate in binding of substrates^12^ (Fig. 1C). The buried catalytic crevice connects with the external environment mainly through the two sides of the L-shaped cavity, further described as Tunnel 1 at the W336 niche side and Tunnel 2 adjacent to the F267 pocket^11^ respectively (Fig. 1D).

In the past decades, a plethora of sEH inhibitors have been generated by prolonged and intense efforts from academia and industry. The majority of these compounds are built around a urea^6,12^, amide^12,13^ and/or carbamate^14^ central pharmocophore disubstituted with hydrophobic moieties^15^, and act *via* an orthosteric mechanism, occupying the active site pocket, thus impeding substrate catalysis^4^. Orthosteric inhibitors of sEH have been an invaluable tool for investigating the biology and biomedicine of sEH^16–18^, filed in hundreds of patent applications, and a few qualified for clinical studies^6,19,20^. Yet, none has to date successfully passed phase 2 clinical trials.

Orthosteric inhibition, however, is not the only mechanism by which the hydrolytic activity of sEH CTD could be opposed. Endogenous electrophilic lipid mediators namely 15-deoxy-Δ^12,14^-Prostaglandin J_2_ (15d-PGJ_2_)^21,22^ and 10-nitrooleic acid decreased blood pressure in an angiotensin-II induced hypertension murine model in an sEH-dependent manner^21,23^. Given that a sEH C521S knock-in mouse model^21,23^ showed resistance to lipid electrophile-mediated vasodilation, mouse C521 (C522 in the human protein) was identified as a key sEH residue covalently targeted by the electrophilic lipids^21,23^ *via* a Michael addition reaction^22^. We further discovered additional cysteine and histidine residues within human sEH CTD which undergo alkylation by 15d-PGJ_2_ and several nitro fatty acids (NO_2_FAs) resulting in enzymatic activity modulation in vitro^22,24^. Notably all these residues are not comprised within the catalytic pocket, invoking allosteric mechanisms of inhibition, which remain uncharacterized to date.

In this study we investigate the allosteric inhibition of sEH CTD at the distal C522 and C423 sites exerted by two ligands identified in previous investigations^22^, specifically 15d-PGJ_2_ and divalent copper ions.

Prostaglandin 15d-PGJ_2_, the dehydration product of prostaglandin D_2_ (PGD_2_), possesses anti-inflammatory effects by acting through PGD_2_ receptors and interacting with a variety of intracellular protein including peroxisome proliferator-activated receptors (PPARs) and sEH^21,22,25,26^. Composed of a carboxyl chain (C1–C7), a cyclopentanone ring (C8–C12) and an aliphatic chain (C13–C20) (Fig. 1E), 15dPGJ_2_ is an α/β-unsaturated ketone electrophilic lipid that reacts in Michael addition reactions with cysteine residues of these proteins, modulating their functions (Fig. 1F). Structural information on 15d-PGJ_2_-sEH complexes featuring the ligand covalently adducted to C522 or C423 could not be obtained, likely because of the dynamic nature of the interaction. In this work we therefore utilized a combination of computational and experimental techniques to elucidate the allosteric inhibitory mechanisms exerted by 15d-PGJ_2_ at these binding sites.

Essential divalent metal ions, including Zn^2+^ and Cu^2+^, were also reported to inhibit sEH activity^27^, providing in principle an additional strategy for modulating the activity of the enzyme^27,28^. Whereas sEH inhibition by Zn^2+^ has been recently investigated in mice^28^, the mechanism by which Cu^2+^ operate remained unclear. Here we present evidence that Cu^2+^ bind to both C423 and C522 sites but only yields a hydrolase inhibitory effect upon C423 association.

Overall, this work revealed the mechanism of allosteric inhibition of sEH CTD at C522 and C423 distal sites, using a combination of computational and experimental techniques. Although the effectors investigated here, 15d-PGJ_2_ and divalent copper ions are structurally unrelated, they converged on the same regulatory cysteine sites, provided an opportunity to compare and rationalize distinct mechanisms of allosteric modulation operating at identical locations within sEH. In line with the theoretical model of the ensemble nature of allostery^29^, we observed that long time scale molecular dynamics and their generated ensembles provided a useful framework for extracting allosteric mechanisms playing a role in regulating sEH. Two distinct mechanisms were hitherto revealed, that differ not only in the allosteric propagation of the signal from the distal to the functional site, but also in the chemical properties of the ligands able to cause inhibition. These highlighted signals and their molecular circuitry provide critical information to be exploited in future pharmacological targeting.

## Results

### REMD simulations reveal distinct conformational states experienced by human sEH when covalently bound to 15d-PGJ₂ *via* C423 and C522

**Fig. 2:**
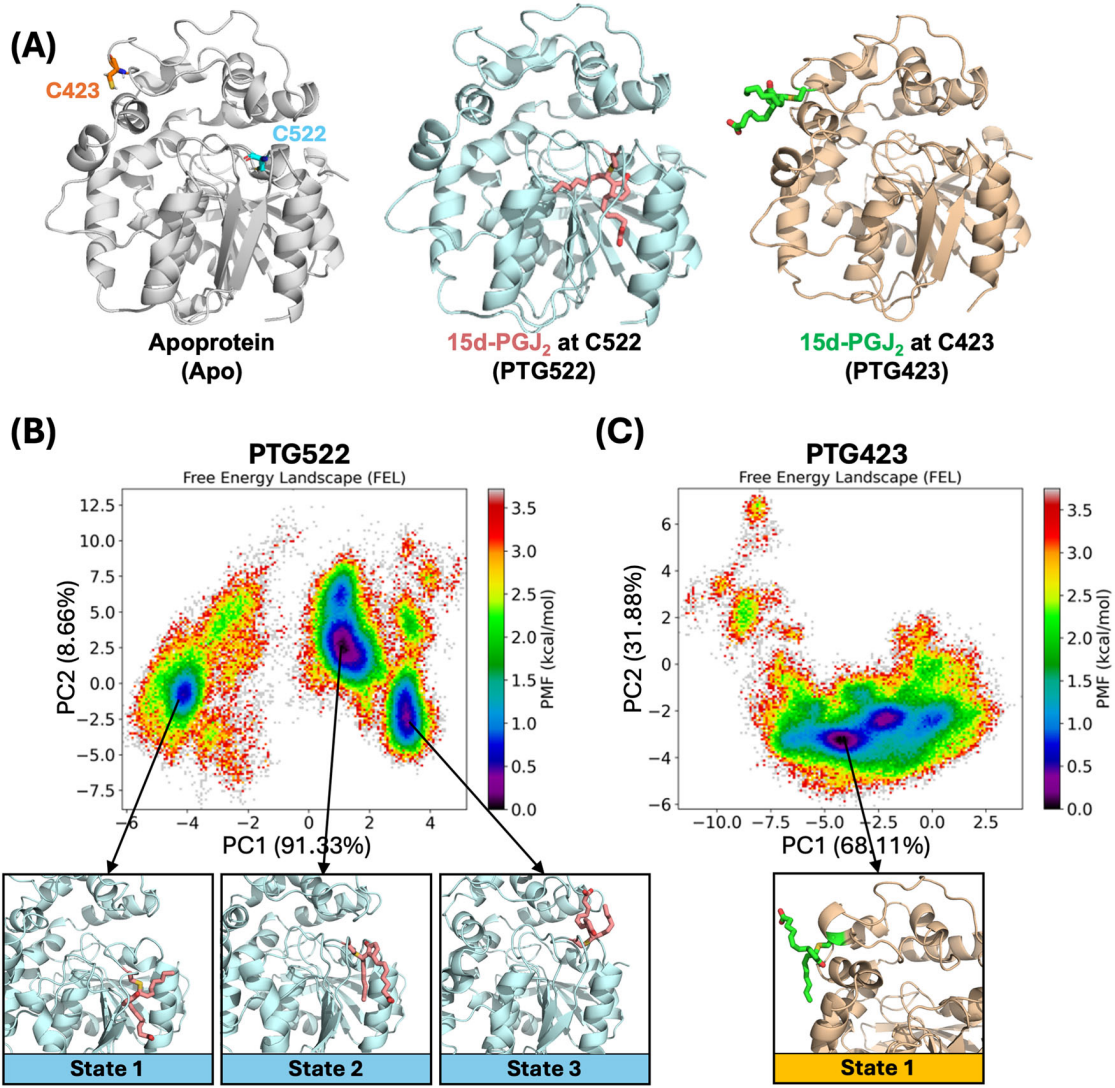
Replica Exchange Molecular Dynamics (REMD) simulation explore new protein–ligand conformations upon 15d-PGJ_2_ binding. **A** Relaxed starting conformations for the three simulation systems: apoprotein (Apo), 15d-PGJ_2_ bound to C522 site (PTG522), and 15d-PGJ_2_ bound to C423 site (PTG423). C522 and C423 are labeled on the apo structure. 15d-PGJ_2_ is shown in sticks and colored in salmon and green for PTG522 and PTG423, respectively. **B** The free energy landscape (FEL) of PTG522 shows three major states (1–3). The relative population of the states 1–3 was calculated as 12.4%, 38.7% and 48.9%, respectively. Representative structures of the three states are shown with sEH CTD displayed in light cyan cartoon and 15d-PGJ_2_ in salmon stick. Potential of mean force (PMF) is colored according to the energy scale. **C** The FEL of PTG423 shows one dominant minimum state (state 1). The representative structure is shown with the protein depicted in wheat cartoon and 15d-PGJ_2_ in green stick.

To investigate the allosteric mechanism of sEH inhibition exerted by 15d-PGJ₂ upon alkylation of residues C423 and C522, 1 μs replica exchange molecular dynamics (REMD) simulations were performed on three systems: the CTD protein alone (apoprotein, Apo), the protein with 15d-PGJ₂ covalently bound to C423 (PTG423), and the protein with 15d-PGJ₂ covalently bound to C522 (PTG522). The relaxed starting structures used for the simulations are shown in Fig. 2A. To assess convergence, the trajectory data was projected onto 2D density maps based on backbone Root-Mean-Square-Deviation (RMSD) (relative to the starting structure) and radius of gyration (Rg) at increasing cumulative simulation times (Supplementary Fig. S1). The density distribution stabilized after 500 ns in all systems, indicating that 1μs REMD simulation was sufficient for further analysis.

Throughout the simulations, several conformations of sEH-15d-PGJ₂ complexes had diverged from the initial covalent docking poses (Supplementary Video S1). To explore and validate these conformational states, we grouped the 15d-PGJ₂ positions using K-means clustering based on the center of mass of the cyclopentanone ring, and visualized the clusters via supervised Principal Component Analysis (PCA) (Fig. 2B, C). Because the PTG423 and PTG522 systems originated from docked structures, which are more prone to inaccuracies, we extended both simulations to 2 μs to ensure that no additional conformational states were missed (Supplementary Fig. S2).

The PTG522 simulations revealed three distinct conformational states. In state 1, the 15d-PGJ₂ lingers near its original docking pose in the core region of sEH CTD (residues 230–348, 494–545), surrounded by β-strands β7 and β8, and by α-helix αF (Fig. 2B). In this state, β-strands β7 and β8 rotate on their respective hinges, uncovering a hydrophobic patch where the alkyl tail of PTG binds (Supplementary Fig. S3). State 2 maps the electrophilic lipid ligand moving away from the core region towards the lid moiety (residues 370–468) and displays a smaller rotation of β7 and β8 compared to state 1. In state 3, the 15d-PGJ₂ molecule protrudes towards the cap-loop (residues 410–443) creating a bridge with the core region and approaching Tunnel 2, one of the channels that connects sEH CTD catalytic pocket with the external environment^11^ (Fig. 2B). No alteration of the β7 and β8 strands was observed in this state.

For the PTG423 system, transient conformations were also observed, but the analysis of the free energy landscape indicated that these were short-lived, without clear distinctive features. Overall. Overall, 15d-PGJ₂ remained extended from C423 towards the solvent, with high flexibility and with a single broad energy minimum (Fig. 2C).

### 15d-PGJ_2_ covalent binding to C522 and C423 modulates sEH tunnel morphology

**Fig. 3:**
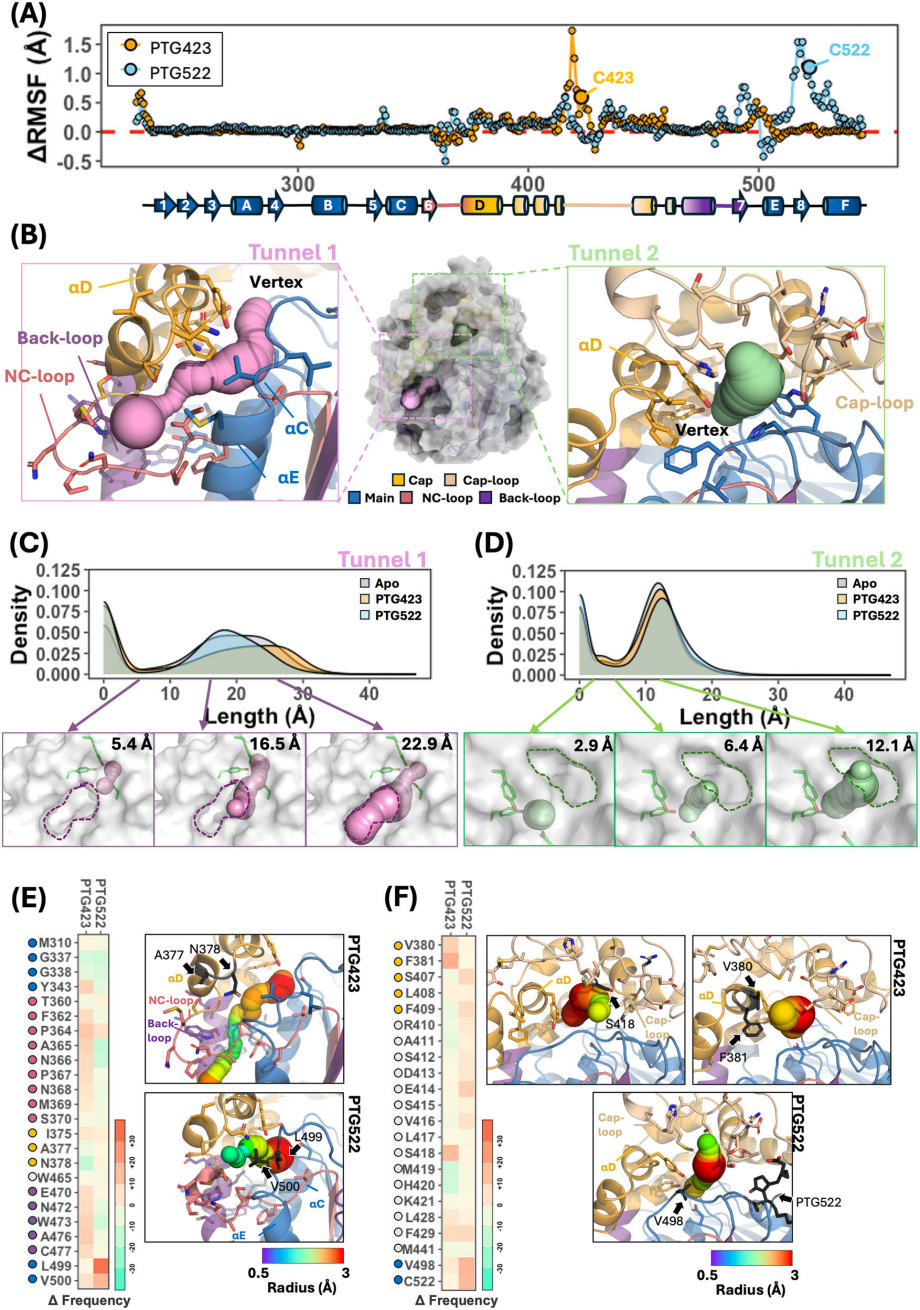
15d-PGJ_2_ binding alters the morphology of sEH tunnels. **A** Per-residue changes in root mean square fluctuation (ΔRMSF) upon 15d-PGJ2 binding. Each dot represents a single residue and C423 and C522 are marked by larger dots. A red dashed line indicates ΔRMSF = 0. A schematic representation of protein secondary structure is reported below residue numbers. **B** Representation snapshots of Tunnel 1 (left, light pink sphere) and 2 (right, green sphere) in the Apo system. Residues lining the two tunnels are shown as sticks. The main, cap, cap-loop, NC-loop and back-loop regions are colored in blue, yellow, wheat, pink and purple, respectively. Length distributions for **C** Tunnel 1 and **D** Tunnel 2 in Apo, PTG423 and PTG522. Representative tunnel images of different lengths are shown with their entrance/exit marked by dashed lines alongside the distribution plot. Changes in tunnel-lining residue frequencies for **E** Tunnel 1 and **F** Tunnel 2 in PTG423 and PTG522 versus Apo. Positive changes in frequencies are in orange and negative are in green. Each reported residue is part of Tunnel 1 (**E**) or 2 (**F**), and is highlighted by a dot colored as their corresponding region depicted in (**B**). Representative snapshots of Tunnels in PTG423 and PTG522 are shown in spheres, key residues are highlighted in black sticks, and tunnel radii are rainbow-colored.

Equipped with the detailed characterization of the conformational equilibria of 15d-PGJ₂-sEH complexes, we next set out to investigate the mechanisms underlying allosteric sEH inhibition by 15d-PGJ₂. Like many enzymes, sEH features a buried active site connected to the outside environment through tunnels, which are responsible for substrate/solvent access and product release^30,31^. Alterations in tunnel morphology can thus impact enzymatic activity. Using CAVER 3.0^32^, we analyzed several parameters concerning Tunnels 1 and 2 in 15d-PGJ₂-bound sEH forms (PTG423 and PTG522) *versus* apo protein (Apo) (Fig. 3).

We first evaluated root mean square fluctuations (RMSF) for PTG423 and PTG522 complexes relative to Apo. In both cases protein flexibility in the NC-loop (residues 349–369) was altered. Moreover, PTG423 induced pronounced changes in the cap-loop (residues 410–443), whilst PTG522 affected the main region (residues 494–545) (Fig. 3A). These protein portions are all critical to both Tunnel 1 and 2: Tunnel 1, which connects the W336 niche, is formed by elements of helices αC/E (main region), helix αD (cap region), the NC-loop and the back-loop (Fig. 3B left); Tunnel 2, towards the F267 pocket, is mainly delineated by the cap-loop and adjacent residues (Fig. 3B right).

Next, we examined tunnel geometry changes. In the various states of the Apo system identified in simulations, Tunnel 1 predominantly exhibited lengths of 15–25 Å measured from the catalytic center defined by residues Y383, Y466, and D335, where lengths above 20 Å would allow solvent access to the sEH vertex (Fig. 3C). Tunnel 2 was typically around 10–20 Å in length, but with some conformations presenting shorter lengths (less than 10 Å). These would make the enzyme catalytic center not easily accessible to solvent (Fig. 3D). Prostaglandin 15d-PGJ₂ binding to sEH modulated tunnel length distributions. Adduction to the C522 site decreased the length of Tunnel 1 to 10–20 Å, while C423 modification extended it (>25 Å) (Fig. 3C); Tunnel 2 length remained mostly unchanged following C522 alkylation but contracted to below 10 Å repeatedly in the PTG423 system (Fig. 3D). This suggests Tunnel 1 or 2 obstruction upon covalent adduction of 15d-PGJ_2_ to C522 and C423, respectively.

To understand better these observed morphological alterations, we analyzed residue participation frequencies in tunnel formation (Fig. 3E, F). In PTG423, an inward shift of the C-terminal end of helix αD was induced, pushing away A377 and N378 from Tunnel 1. This conformational rearrangement redirects and elongates the tunnel underneath the NC-loop, and this is accompanied by an increased participation of NC-loop and back-loop compared to Apo in tunnel formation (Fig. 3E). On the other hand, Tunnel 1 was considerably shortened in PTG522, primarily through movement of L499 and V500, which occlude the tunnel midsection and narrow its radius (Fig. 3E). Tunnel 2 showed slight disruption in PTG423 compared to Apo, mediated by an increase of cap-loop flexibility and αD displacement. Cap-loop residues 410–417 and 419–420 showed reduced participation, while new amino acids such as V380 and F381 (αD) and S418 (cap-loop) were identified to form the lining of Tunnel 2 (Fig. 3F). This geometry change would cause tunnel obstruction (Fig. 3F). In contrast, PTG522 preserved overall tunnel architecture, with modest increases in involvement of the cap-loop and nearby residues such as V498 (Fig. 3F).

Collectively, these findings suggest that 15d-PGJ₂ covalent modification at the two allosteric sites causes sEH tunnel remodeling in a site-specific manner: adduction at C423 seems to alter Tunnel 2 properties through involvement of the upper cap-loop and cap (αD) regions, while binding at the C522 site predominantly appears to alter Tunnel 1 through affecting the lower main (αE) and NC-loop regions.

### AlloHubPy identifies distinct allosteric communication pathways upon 15d-PGJ₂ covalent modification at C423 and C522

Allosteric effects can originate from changes in the internal dynamic communication between distal allosteric sites and the catalytic pocket^29,33^. We therefore investigated how 15d-PGJ_2_ distal binding to C522 and C423 influences the active site of sEH by employing AlloHubPy, an information-theory based tool that detects changes in interaction patterns using MD simulations and a structural alphabet approach^34^. AlloHubPy segments the protein into overlapping four-residue fragments (see “methods”), encoding them as structural alphabet elements. Correlated motions between fragments are then analyzed to infer communication pathways. We applied AlloHubPy to Apo, PTG423, and PTG522 systems, performing a comparative analysis to evaluate the effect of 15d-PGJ_2_ association on the internal signaling of the protein.

**Fig. 4:**
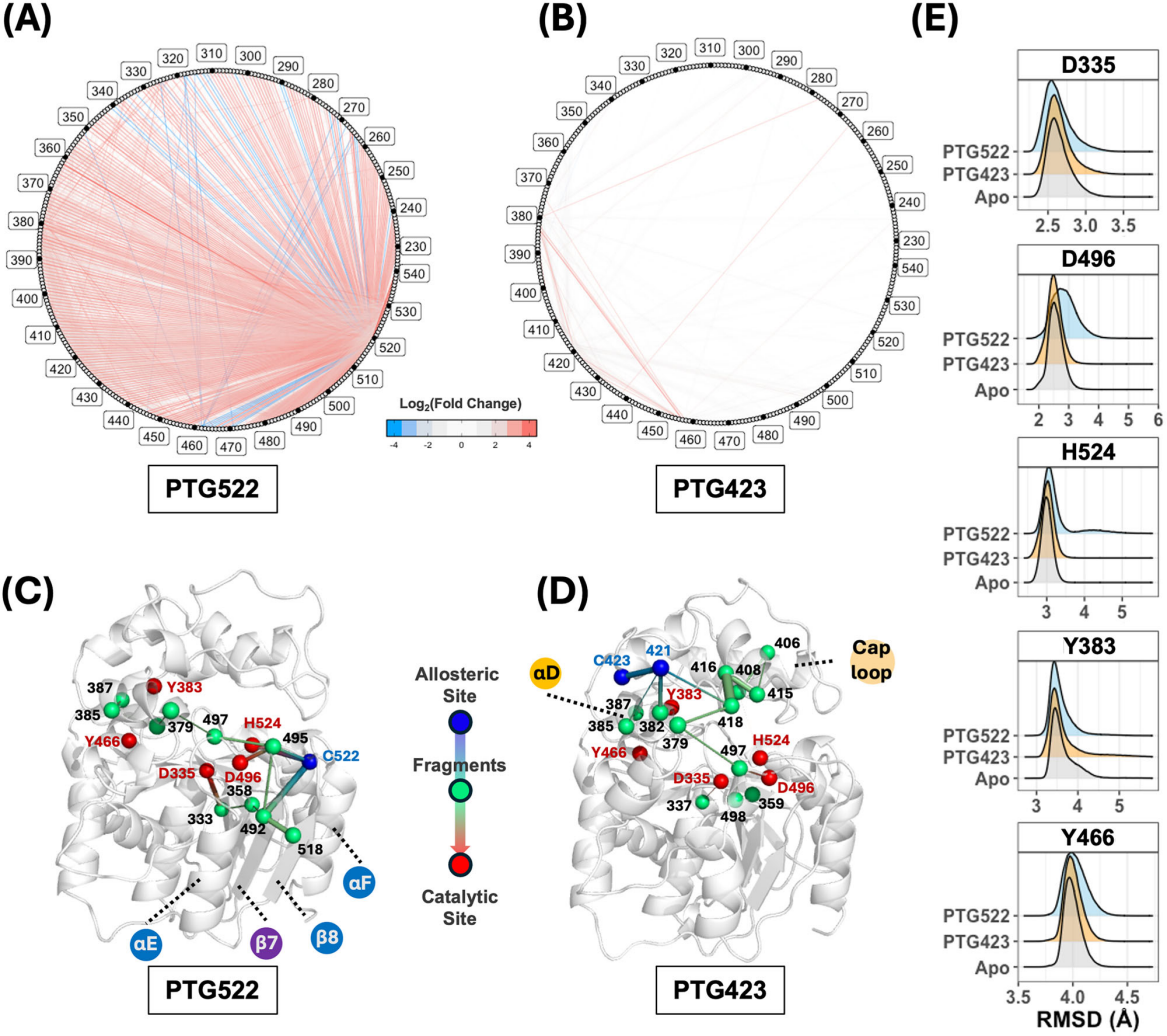
AlloHubPy reveals allosteric communication paths to the catalytic center. Log_2_ fold-change in fragment signal coupling in **A** PTG522 and **B** PTG423, compared to Apo. Fragments 229–546 are shown as dots on a circular layout; black dots mark every 10 fragments. Red and blue lines indicate increased or decreased correlations between two fragments respectively. The shortest communication pathway connecting allosteric site (blue) through intermedia fragments (green) to catalytic residues (red) in **C** PTG522 and **D** PTG423 are calculated from the net change in signal coupling. Line thickness reflects coupling strength; key regions are labeled and outlined with dashed lines. **E** Root-Mean-Square-Deviation (RMSD) distribution of catalytic residues (D335, D496, H524, Y383, Y466) relative to the average apoprotein conformation in systems Apo, PTG423, and PTG522.

With these analyses, net changes in fragment coupling induced by ligand binding were mapped (Fig. 4A-B). Covalent adduction of 15d-PGJ₂ at C522 resulted in broader and stronger coupling alterations compared to C423. In the PTG423 system, fragment 418 (residues 416–420) showed the most notable changes (Fig. 4B), suggesting its role as a communication hub following 15d-PGJ₂ interaction. Fragment 418 had increased coupling with several other fragments in the lid region, in particular 382 (residues 380-384) which is near the epoxide positioner Y383. These residues lie in the cap-loop and αD region, consistent with our tunnel analysis where PTG423 modulates Tunnel 2 formation *via* steric hindrance involving S418, V380, and F381 (Fig. 3F).

In the PTG522 system, fragment 518 (residues 516–520), located on the hinge of β8 and adjacent to C522, exhibited significantly enhanced coupling with several distant fragments, including 362 (residues 360-364) on the NC-loop, a region contributing to Tunnel 1 formation (Fig. 3B). This communication pattern suggests that tunnel remodeling and catalytic inhibition upon C522 adduction may be mediated by core domain signaling through fragment 518. Notably, analysis across individual conformational states of PTG522 showed consistent communication patterns, with a slight attenuation in state 3, where the conformational change is not observed (Supplementary Fig. S4).

To map the propagation of allosteric signals to the catalytic center, we identified the shortest communication pathways from the allosteric sites (C423 or C522) to key residues of the sEH vertex (Y383, Y466, D335, D496, H524). Internal communication networks were first constructed for each system separately (Supplementary Fig. S5). This analysis revealed that the reaction of 15d-PGJ_2_ with C522 initiated new pathways involving fragments in the protein core domain, particularly fragment 492 and 518 which map to the conformationally altered β7 and β8 strands, respectively. In contrast, the binding event at C423 primarily enhanced pre-existing routes observed in the apoprotein (Apo) connecting C423 to the catalytic vertex *via* the cap-loop (Supplementary Fig. S5). The shortest signaling paths computed based on net coupling changes further confirmed these trends (Fig. 4C, D). In the PTG522 system, allosteric communication propagated from the distal site (C522) to the catalytic triad residues D496 and H524 *via* fragments in the protein core region. Fragment 492 and 518 acted as key elements, suggesting their potential regulatory impact on the catalytic center (Fig. 4C). Instead, for PTG423 signal transmission occurred largely onto the epoxide positioner Y383 on αD. Enhanced signaling was also detected through the cap-loop (fragments 418, 416, 415; residues 413–420) (Fig. 4D), consistent with Tunnel 2 obstruction observed by CAVER (Fig. 3F).

We next examined the structural perturbations experienced by sEH key catalytic residues in the 15d-PGJ₂ adducted complexes by computing their RMSD relative to the average apoprotein structure. Interaction of 15d-PGJ₂ at C522 site increased the RMSD of both D496 and H524, while ligand binding at C423 specifically perturbed Y383, consistent with the observed allosteric signaling by AlloHubPy (Fig. 4E).

Taken together, the integration of tunnel morphology and AlloHubPy analyses reveals the presence of a dual allosteric mechanism for sEH: binding at the C423 site perturbs the upper lid (cap and cap-loop), obstructing Tunnel 2 and affecting Y383, while C522 binding alters the lower protein core domain (main and NC-loop regions), mainly through a rearrangement of β7 and β8, impacting on Tunnel 1 as well as catalytic residues D496 and H524.

### Ligand-protein contact analysis highlights different ligand conformation requirements for C423 and C522

**Fig. 5:**
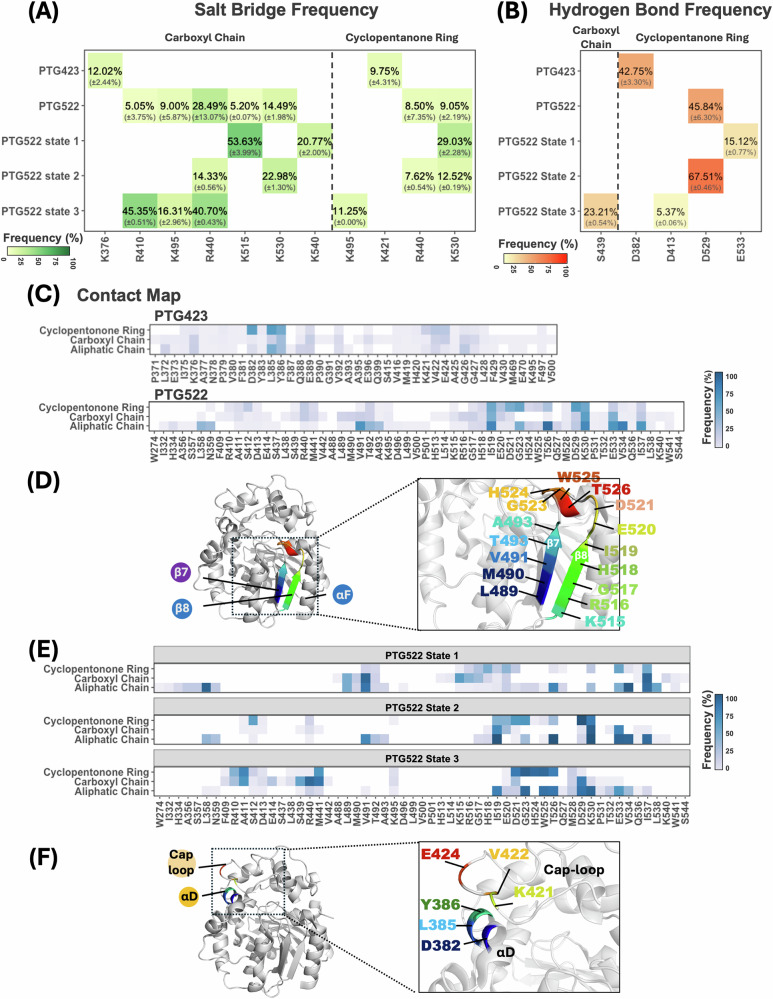
Protein–ligand interactions analysis of 15d-PGJ_2_ with sEH CTD in most representative conformational states extracted from simulations. **A** Frequency of salt bridge formation between 15d-PGJ_2_ (carboxyl group or cyclopentanone ring) and sEH CTD lysine or arginine residues, calculated using a distance cut-off of 4.5 Å (mean ± standard deviation (SD)). Only interactions occurring with a frequency greater than 5% are displayed. **B** Hydrogen bonding frequencies (≥5%) between sEH and the cyclopentanone ring or carboxyl chain of 15d-PGJ_2_ as determined using MDAnalysis (mean ± SD). **C** Contact maps of sEH with each 15d-PGJ_2_ moiety (cyclopentanone ring, carboxyl chain, and aliphatic chain) calculated by an inter-atom cut-off of 4 Å. This includes hydrophobic/van der Waals type of contacts. **D** Structural details showing residues with frequent contacts to 15d-PGJ_2_ in PTG522, colored as rainbow. **E** Contact map of the three major conformational states of PTG522. **F** Structural details of key interacting residues in PTG423, colored as rainbow.

To gain insight into which atomic-level interactions established by the prostaglandin 15d-PGJ₂ would account for the observed allosteric effects, we monitored ligand-protein contacts throughout the simulations; an overall contact map, alongside an analysis of salt bridges and hydrogen bonds is shown in Fig. 5.

In the PTG522 system, 15d-PGJ_2_ established multiple interactions with sEH. Specifically, its carboxyl chain formed salt bridges with residues R440, K495 and K530 (for 28.49%, 9.0% and 14.49% of simulation time respectively), the cyclopentanone ring engaged in hydrogen bonding with D529 (45.84%), and the carboxyl chain interacted with S439 via hydrogen bonding (3.94%) (Fig. 5A, B). Additionally, both the cyclopentanone ring and aliphatic chain established multiple contacts with amino acids 491-493 and 515-537 (Fig. 5C). Notably, these residues are located on strands β7, β8 and helix αF within the core region of sEH (Fig. 5C, D), adjacent to fragments 492 and 518 identified in allosteric signal propagation by AlloHubPy (Fig. 4C).

Examination of ligand-protein interaction patterns in each of the three PTG522 states taken individually revealed key differences. Overall, fewer salt bridges and hydrogen bonds between 15d-PGJ_2_ and sEH were detected in states 1 and 2, contrary to state 3 where longer-lived polar interactions were observed, particularly with residues R410, R440, K495 and S439 located on or nearby the cap-loop region (Fig. 5A, B). Such ligand-protein contacts may account for state 3 being the most populated in REMD simulations; however, despite stronger polar contacts, state 3 exhibited the weakest allosteric communication by AlloHubPy (Supplementary Fig. S4). This could be linked to the comparative contact patterns of the three states, as well as the observed local conformational change of strands β7 and β8 in states 1 and 2. While state 3 established contacts with residues 521–526 (spanning the linker between β8 and αF), state 1 and 2 engaged with residues 489–493, 515–526 (β7, β8 and linker between β8 and αF) and 491–493, 519–526 (end of β7, end of β8 and linker) respectively (Fig. 5D-E), thus closer to the fragment hubs 492 (residues 490–494 on β7) and 518 (residues 516–520 on β8) revealed by AlloHubPy to play important roles in allosteric communication.

In contrast to PTG522, the PTG423 system showed fewer 15d-PGJ₂-sEH interactions (Fig. 5A–C), highlighting a distinct interaction regime at this site. These contacts included a salt bridge between the carboxyl chain of the prostaglandin and K376 (for 12.02% of simulation time) and a hydrogen bond between the cyclopentanone ring and D382 on αD (42.75%) (Fig. 5A, B). Hydrophobic/van der Waals contacts are also limited, involving predominantly the cyclopentenone ring of the lipid and protein residues D382, L385 and Y386 on αD and K421, V422, E424 on the cap-loop (Fig. 5C, F). The relative scarcity and peripheral nature of these ligand–protein contacts suggest that, in contrast to PTG522, the inhibitory allosteric effect at C423 may originate predominantly from the local covalent interaction with cysteine 423.

### Experimental mutagenesis of sEH alters the 15d-PGJ_2_ inhibitory effect at C522

Our REMD and AlloHubPy results indicate three distinct conformational states for sEH CTD-15d-PGJ₂ complex adducted at C522. Among these, state 3 emerged as the most populated, exhibiting a distinctive protein-ligand interaction profile compared to states 1 and 2, yet the weakest in terms of overall allosteric coupling to sEH catalytic center. This combination of high population and mechanistic distinction made state 3 an ideal candidate for mutagenesis aimed at testing its functional relevance.

**Fig. 6:**
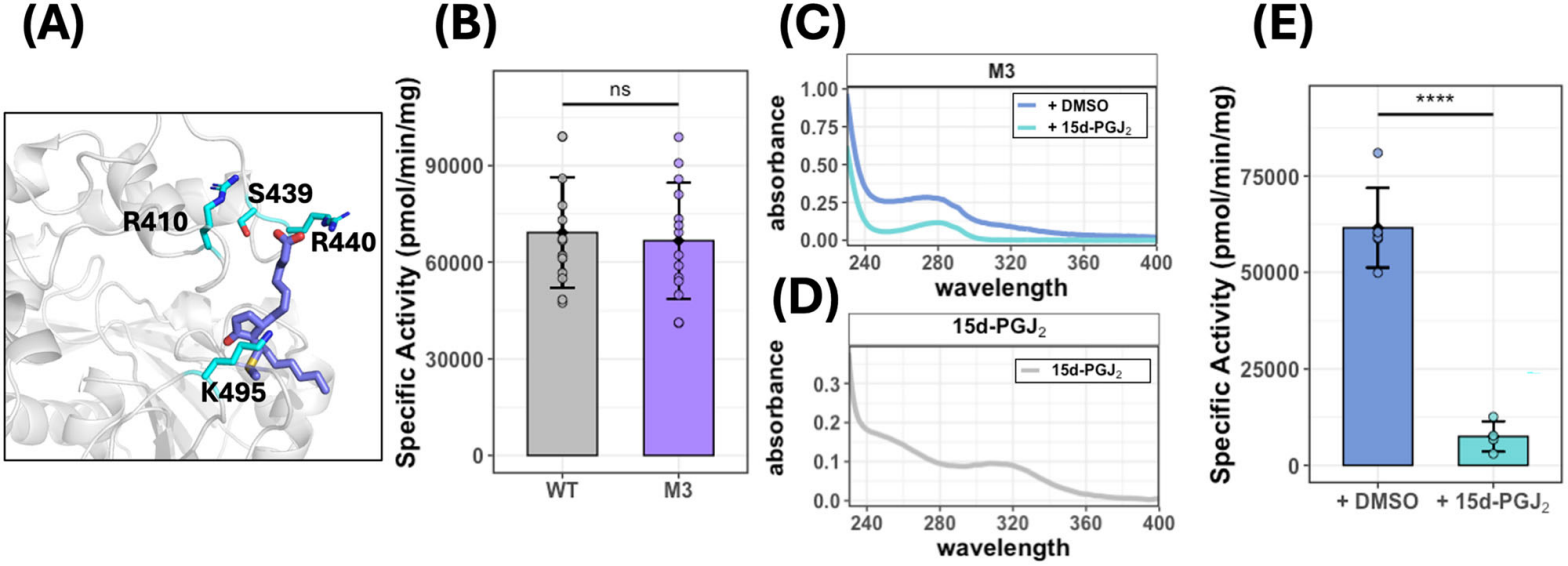
Mutagenesis affecting state 3 alters the inhibitory effect at C522 site. **A** Visualization of sEH residues that form salt bridges and hydrogen bonds with 15d-PGJ_2_ in state 3 of PTG522. These residues are mutated in M3 mutant. **B** Enzymatic activity assay of mutant M3 (C423S/R410A/S439A/R440A/K495A) compared to WT, obtained from *n* = 13 biological replicates. No difference was observed. Circles and error bars indicate mean and standard deviation (SD). (C-D) UV spectra of **C** M3 reaction mixture with DMSO or 15d-PGJ_2_, compared with the UV spectrum of **D** 15d-PGJ_2_ alone. **E** Enzymatic specific activity assay following treatment of M3 with DMSO or 15d-PGJ_2_. obtained from *n* = 4 biological replicates. Circles and error bars indicate mean and SD. Statistical significance was performed by one-way ANOVA followed by Tukey’s HSD test: ns (*p* > 0.05, not significant), * (0.01 < *p* ≤ 0.05), ** (0.001 < *p* ≤ 0.01), ***(0.0001 < *p* ≤ 0.001), **** (*p* < 0.0001).

We therefore set out to alter the relative population between the different states revealed by REMD. We reasoned that mutating sEH residues involved in stabilizing state 3 would disrupt these direct ligand-protein interactions, shifting the conformational equilibrium away from state 3. Based on their salt bridges and hydrogen bond contacts with the carboxyl chain of 15d-PGJ_2_ in state 3, R410, S439, R440 and K495 were selected for mutagenesis (Fig. 5A, B; Fig. 6A). Additionally, a C423S substitution was introduced to ensure that 15d-PGJ_2_ would only modify C522.

The resulting mutant sEH CTD M3 (C423S/R410A/S439A/R440A/K495A) was first subjected to circular dichroism (CD) (Supplementary Fig. S6) and enzymatic activity analyses (Fig. 6B) to ensure that these mutations did not impair protein structure and function. M3 was then incubated with a 15-fold molar excess of 15d-PGJ_2_. After extensive washing to remove any non-covalently bound ligand, a covalently adducted sEH-15d-PGJ_2_ species was obtained, confirmed by the 15d-PGJ_2_ characteristic absorbance peaks at 250 and 306 nm^22^ in the UV spectra (Fig. 6C, D).

Our experiments showed that 15d-PGJ_2_ adduction to C522 in the M3 mutant resulted in an 8.2-fold reduction in enzymatic specific activity relative to the DMSO control (Fig. 6E). By comparison, our previous work reported that 15d-PGJ_2_ covalent binding to C522 (in the C423S mutant) caused only a 2.2-fold inhibition^22^. The enhanced potency exerted by adduction to C522 in the context of the M3 mutant suggests that disrupting state 3 amplifies the functional impact of 15d-PGJ_2_ on sEH CTD hydrolytic activity. This observation suggests that state 3 has little (if any) allosterically activity, whilst also validating our REMD and AlloHubPy results showing that state 1 and 2 generate stronger allosteric coupling linked to inhibitory effects through protein-ligand contacts.

### C423 is a permissive allosteric site in human sEH

Previous studies demonstrated that sEH could be blocked by the divalent copper metal ion^27^. The quest to find alternative therapeutic strategies to block sEH led us to investigate this mechanism of sEH CTD inhibition. Serendipitously, these experiments served to support our in silico derived hypothesis of different mechanisms of allosteric inhibitions mediated by the C522 and C423 sites, as well as giving insights into the ligand properties required to perform this function.

**Fig. 7:**
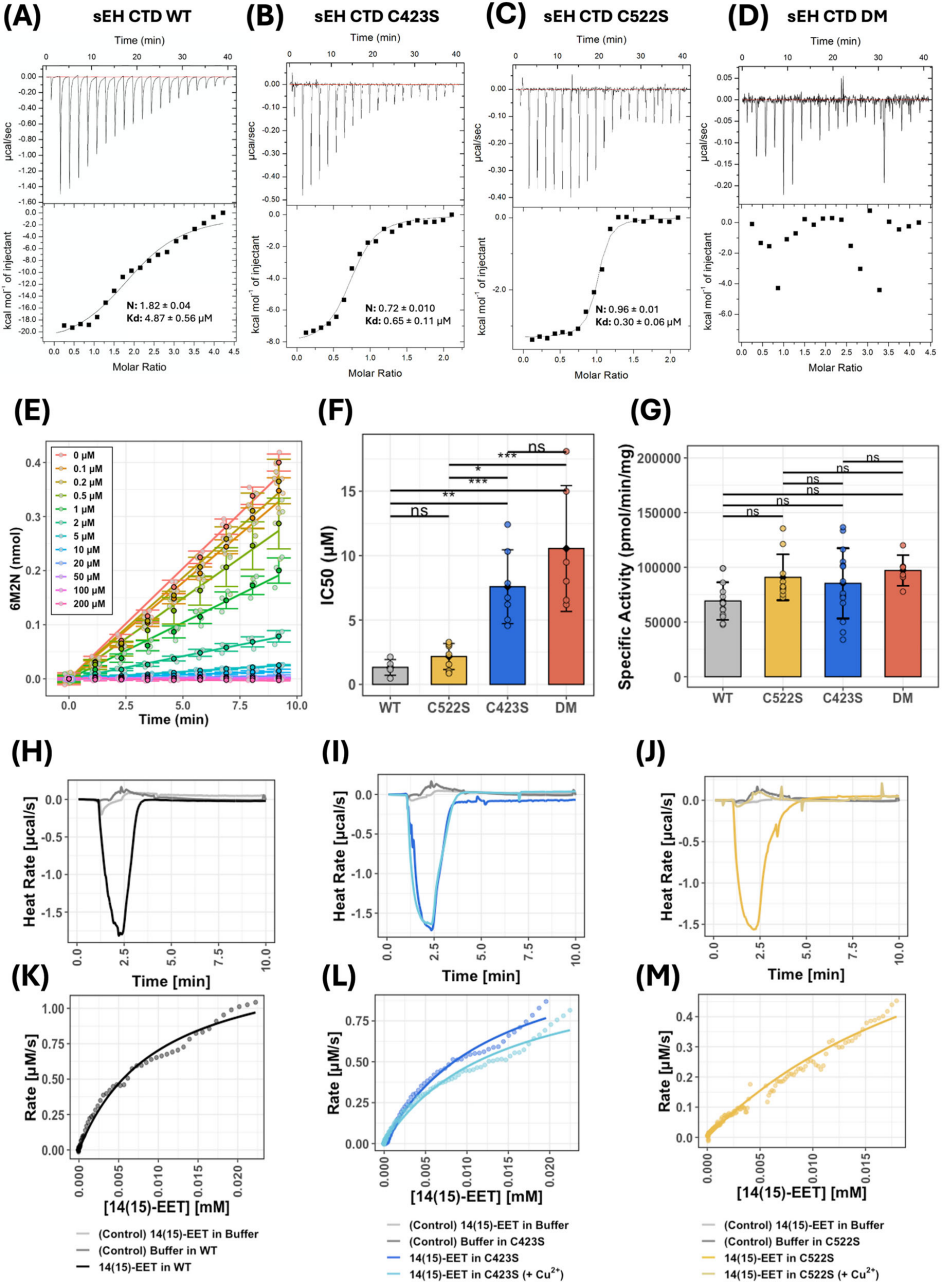
Divalent copper metal ions inhibit sEH *via* C423 but not C522. ITC experiments showing the thermal effect of mixing Cu^2+^ with **A** WT, **B** C423S, **C** C522S and **D** Double Mutant (DM, C423S/C522S). Molar ratio (N) and dissociation constants (*K*_D_) are calculated using *n* = 3 independent biological replicates. For each interaction a representative raw data and normalized binding curve is shown. Black dots represent the normalized heat obtained at each injection and the gray curves show the best fit obtained by a non-linear least-square method based on independent binding site model. ITC experiments show that Cu^2+^ association with sEH involves the two allosteric sites C423 and C522. **E** Graphs of enzymatic activity assays conducted in the presence of increasing concentrations of Cu^2+^ showing inhibition of sEH CTD WT. Individual data points are shown as colored circles corresponding to each Cu^2+^ concentration, while mean values are shown as black-outlined color-filled circles (color coded to the corresponding Cu^2+^ concentrations); error bars represent the standard deviation (SD). **F** IC50 values calculated from enzymatic activity assay experiments for WT, C522S, C423S and DM, using *n* = 5, 7, 7, and 6 independent replicates, respectively. C522 mutation does not alter the WT IC50 value, contrary to C423S and DM. Circles and error bars indicate mean and SD. **G** Enzymatic activity assay showing that all the proteins (WT, C522S, C423S, DM) have comparable enzymatic specific activity, derived from *n* = 13, 10, 16, and 6 biologically independent replicates, respectively. Circles and error bars indicate mean and SD. Statistical significance was performed by one-way ANOVA followed by Tukey’s HSD test; ns (*p* > 0.05, not significant), * (0.01 < *p* ≤ 0.05), ** (0.001 < *p* ≤ 0.01), ***(0.0001 < *p* ≤ 0.001), **** (*p* < 0.0001). **H**–**J** Representative single-injection-method (SIM) ITC thermal profiles and **K**–**M** their corresponding Michaelis-Menten kinetics curve fitting obtained titrating 14(15)-EET into sEH CTD protein **H**, **K** WT, **I**, **L** C423S, and **J**, **M** C522S in the presence or absence of Cu^2+^. Curves are color-coded as indicated in each of the panels. SIM-ITC experiments were performed in *n* = 3 independent biological replicates; derived kinetics parameters are provided in Table 2.

Binding experiments carried out using isothermal titration calorimetry (ITC) revealed that Cu^2+^ interacts with sEH CTD WT with a dissociation constant (*K*_D_) of 4.87 ± 0.56 μM and a ligand-to-protein molar ratio of around two – in other words, two Cu^2+^ binding sites per CTD molecule (Fig. 7A, Table 1). Substitution of each of the two allosteric sites C423 and C522 to serine (namely C423S and C552S respectively) halved the stoichiometry of binding in both cases (Fig. 7B, C, Table 1), while the double mutation (DM; C423S/C522S) abolished detectable association, indicating that Cu^2+^ binds to sEH CTD on two distinct sites involving cysteine C423 and C522 respectively (Fig. 7D).

**Table 1:** Thermodynamics parameters of sEH CTD–Cu^2+^ association measured by isothermal titration calorimetry (ITC)

Protein	ΔH (kcal/mol)	N (Ligand:Protein)	*K*_D_ (μM)	-TΔS (kcal/mol)	ΔG (kcal/mol)
WT	−9.59 ± 0.27	1.82 ± 0.04	4.87 ± 0.56	5.51 ± 9.20	−7.20 ± 0.07
C423S	−8.55 ± 0.17	0.72 ± 0.01	0.65 ± 0.11	0.28 ± 0.53	−8.20 ± 0.09
C522S	−3.53 ± 0.05	0.96 ± 0.01	0.30 ± 0.06	−5.00 ± 1.90	−8.76 ± 0.12
DM	N.D.	N.D.	N.D.	N.D.	N.D.

(Mean ± standard deviation; *N.D*. not detectable). These were calculated from *n* = 3 biologically independent replicates.

The effect of Cu^2+^ association onto the allosteric sites C522 and C423 of sEH was investigated through IC50 measurements^22,24^ (Fig. 7E, F). This showed that increasing concentrations of Cu^2+^ were accompanied by a decrease of sEH CTD WT hydrolytic activity, with an IC50 of 1.32 ± 0.61 μM (Fig. 7E, F). Notably, whereas the C522S mutant measurements largely recapitulated the behavior of CTD WT (Fig. 7F), the IC50 values in the presence of Cu^2+^ were considerably higher for the C423S mutant, and comparable to the double C522S/C423S mutant (Fig. 7F).

These experiments indicate that sEH CTD is enzymatically inhibited by divalent copper ions and, although Cu^2+^ binds to both allosteric sites around C423 and C522, only the association with the former results in enzyme inhibition. Of note, the specific activity of all mutants was not significantly altered compared to WT (Fig. 7G), validating that the observed inhibitory effects are attributable to Cu^2+^ association rather than inherent enzymatic activity differences between the protein variants.

To further characterize the inhibition exercised by sEH-Cu^2+^ association on and around C423, enzymatic kinetics measurements were performed employing an ITC single injection method (SIM) previously developed by our group^35^. We used the *par excellence* sEH CTD substrate, the 14(15)-epoxyeicosatrienoic acid (EET) (Fig. 7H–M, Supplementary Fig. S7). The experiments with sEH CTD WT gave a turnover rate (k_cat_) and a substrate affinity (K_M_) of 5.41 ± 0.10 s^−1^ and 11.17 ± 0.46 μM, respectively, consistent with previous literature^35^ (Fig. 7H, K, Table 2). No statistically significant change was observed for C423S and C522S mutants (Fig. 7I–M, Table 2). In presence of Cu^2+^ ions the sEH-mediated catalysis of 14(15)-EET remained unaltered for C423S (Fig. 7I, Table 2), while no hydrolysis of 14(15)-EET could be detected for C522S (Fig. 7J, Table 2).

**Table 2:** Kinetics parameters for sEH CTD hydrolysis of substrate 14(15)-EET measured by isothermal titration calorimetry (ITC) single injection method (SIM)

Protein	ΔH (kcal/mol)	*k*_cat_ (s^−^^1^)	*K*_M_ (μM)	*K*_sp_ (s^−^^1^ μM^−^^1^)
WT	−9.41 ± 1.12	5.41 ± 0.10	11.18 ± 0.46	0.48 ± 0.02
C423S	−9.61 ± 1.27	4.72 ± 0.08	14.57 ± 0.52	0.32 ± 0.01
C423S:Cu^2+^ (1:2)	−8.73 ± 0.43	4.87 ± 0.10	12.47 ± 0.47	0.39 ± 0.02
C522S	−8.65 ± 0.81	3.79 ± 0.27	23.96 ± 2.58	0.16 ± 0.02
C522S:Cu^2+^ (1:2)	N.D.	N.D.	N.D.	N.D.

(Mean ± standard deviation; *N.D*. not detectable). These were calculated from *n* = 3 biologically independent replicates.

Together, these experiments reveal that Cu^2+^ can inhibit sEH CTD hydrolytic activity by interaction with the allosteric site C423. Cu^2+^ binding to C522 has instead limited (if any) inhibitory effect. This endorses our in silico results implicating the cyclopentanone ring as well as the carboxyl and aliphatic chains of 15d-PGJ₂ in the allosteric mechanism of inhibition at the C522 site. The inability of Cu^2+^ to inhibit through C522 reinforces the notion that this site depends on the ligand’s characteristics for productive allosteric inhibition. Conversely, C423 appears to serve as a more permissive allosteric site, capable of accommodating a variety of ligands such as 15d-PGJ₂ as well as divalent metal ion Cu^2+^.

## Discussion

The hydrolytic activity of sEH has been identified as an important modulator in cardiovascular, pulmonary, renal and brain health^2,3,7,36–38^. Inhibitors of sEH offer broad therapeutical protection, including reduction in hypertension and atherosclerosis, prevention of heart failure, regression of inflammation, neurological disorders and fibrosis, as well as analgesia^2,3,5,7,17,36,39^. Failures in clinical trials of orthosteric compounds underscored the need for alternative pharmaceutical approaches to target sEH CTD, less reliant on orthosteric pharmacophores. One of such strategies would envisage harnessing the potential of covalent allosteric effectors for therapeutic inhibition.

In general terms, covalent allosteric inhibition has gained increasing attention as a promising avenue for modulating protein function in several systems beyond sEH^40^. For example, the SUMO E1 enzyme is allosterically inhibited by COH000 through covalent Michael addition at cysteine C30^41^, Akt kinase is targeted by Borussertib at allosteric sites C296 and C310^42,43^, cucurbitacin B inhibits PCK2 by modifying C63^44^, and 9-nitro-oleic acid (9NO_2_OA) blocks FBPase via the distal C290 site^45^. Within the α/β-hydrolase superfamily, *Arabidopsis* epoxide hydrolase isoform 7 (AtEH7, AT4G02340.1) is blocked by the pan-HER kinase drug Neratinib through covalent attachment at a surface-exposed cysteine (C186) in its cap-loop^46^, spatially resembling C423 of human sEH investigated in this study. These examples not only establish the broader relevance of covalent allosteric inhibition across diverse enzyme families but also highlight reactive cysteines as druggable features. Indeed, several covalent drugs featuring electrophilic warheads that target cysteines, such as afatinib, ibrutinib and osimertinib, have been approved by the US Food and Drug administration (FDA), providing successful examples of this emerging class of covalent therapeutics^47–49^.

Concerning sEH, previous work demonstrated that its hydrolytic activity can be endogenously impaired by covalent adduction of electrophilic lipids—including 15d-PGJ_2_, 9-nitro-oleic acid (9NO₂OA), 10-nitro-oleic acid (10NO₂OA) and 10-nitro-linoleate (10NO₂LA)—onto the allosteric distal sites C522 and C423^21–24^. In this study, we characterize the allosteric phenomena at these residues through investigation of 15d-PGJ₂ and copper divalent ions. The integrated computational and experimental results determined that C423 and C522 differ in their allosteric mechanism and ligand-binding properties, with each site exhibiting distinct effector binding requirements for effective inhibition.

For 15d-PGJ₂, in silico analyses revealed site-specific perturbations of protein structure and transmission pathways from the distal sites to the catalytic cleft. Modification at C522 was found to alter Tunnel 1 through a conformational rearrangement of the residues in the protein lower core region, whereas conformational changes in the upper lid region affecting Tunnel 2 morphology were traced upon C423 binding. These tunnel alterations likely compromise substrate access and gate solvent entry to the active site, consistent with the allosteric nature of inhibition. In terms of allosteric signaling, we revealed that the binding event of 15d-PGJ₂ at C522 activated communication networks in the protein core region largely through fragment 492 (residues 490–494) and 518 (residues 516–520), propagating to catalytic residues H524 and D496. In contrast, C423 adduction elicited modulation of the epoxide positioner Y383, as well as perturbing the cap-loop region, a portion of the protein that participates in Tunnel 2 shaping and is known to influence substrate selectivity in epoxide hydrolases^10,11^.

Conformational ensemble and ligand–protein contact analyses provided further insights into the mechanism underlying these allosteric modulations at the atomistic level. Through its cyclopentanone ring, aliphatic and carboxyl chains, C522-bound 15d-PGJ₂ establishes extensive interactions with sEH spanning from the core to the lid region, stabilizing one or more of three sampled conformational states. Of these, the apolar contacts with the protein lower core seen in states 1 and 2 play key roles, likely producing a displacement of the strands β7 and β8, impacting on Tunnel 1 substrate accessibility potential and disseminating the allosteric signaling through the structure to the catalytic site. A complementary experimental approach supports these findings, showing that mutations disrupting the less allosterically active (if not inactive) state 3 enhance the allosteric inhibition potency of 15d-PGJ₂ at the C522 site. This increase is likely linked to a redistribution of the conformational ensemble, in which removal of the less active state raises the population of the remaining clusters, both characterized by a distinct conformational fingerprint.

Taken together, these results highlight the importance of analyzing quantitatively multiple conformational ensembles derived from simulations to uncover functional mechanisms playing a role in allosteric regulation. Notably, our findings conclusively establish that the mechanism of inhibition by electrophilic lipids *via* the C522 site is indeed an allosteric response rather than a steric hindrance effect which was hypothesized given the C522 location at the exit of the catalytic channel^50^.

Contrary to C522, 15d-PGJ₂ adduction at C423 yields a single dominant conformation and enlists fewer ligand-protein contacts. Intriguingly, binding of divalent copper ions at C423 led to hydrolytic inhibition of sEH whilst association around the C522 site did not, as appraised using a variety of experimental approaches. In addition to uncovering new allosteric effectors for sEH, these experiments are consistent with the in silico findings revealing the key contribution of 15d-PGJ₂ chemical interactions to productive allosteric inhibition at C522, but not C423. In other words, allostery at C522 would depend on the chemical nature of the effector, whereas the C423 site appears more permissive and tolerant to ligand variability to generate an allosteric response by diverse agents. Noteworthy, this newly discovered structural and dynamic mechanisms of allostery as well as the diverse features of the effectors at the two C423 and C522 sides offer new exciting opportunities for drug design^51,52^. Targeting C522 may allow for fine-tuned, structure-guided design of covalent inhibitors to optimize binding affinity, adduction capability and allosteric modulation of sEH CTD activity. Notably, the next candidates for such a structure-activity relationship campaign already exist, the experimentally validated NO_2_FAs^23,24^. Conversely, engaging C423 may be advantageous for expanding the search to modulators with different chemical scaffolds and properties, implementing the latest frameworks for computational design of allosteric effectors^51,52^.

Finally, the allosteric inhibition exerted by Cu^2+^ onto C423 revealed a hitherto unknown form of metalloallostery^53^ for sEH. Although copper has recently been identified and characterized as an allosteric modulator of protein function, the dynamic metalloproteome is likely to be underestimated and new copper-dependent proteins are expected to be revealed^53^. Because of the transient interactions mediated by non-canonical coordination motifs in metalloallostery, computer prediction tools that work for traditional metal-binding proteins have been ineffective for the identification of metalloallostery sites^53^. In line with this, BioMetAll and other software were unable to identify C423 and C522 in their top-rank prediction^54^. Our findings that sEH hydrolytic activity could be negatively modulated by Cu²⁺ in vitro will pave the way for further investigations of its functional relevance, which could potentially be of medical interest given that copper deficiency and/or overload have been correlated with onset and development of many pathological conditions, including obesity, fatty liver disease, neurodegeneration, cancer and inflammation^53,55–58^.

In conclusion, this work provides a fundamental understanding of how the hydrolytic activity of sEH is regulated by covalent post-translation modifications as well as metal ions. Furthermore, this study adds to the growing understanding of cysteine-based allostery as a flexible and evolvable mode of enzyme regulation and points to new strategies for therapeutic development leveraging endogenous lipid electrophiles and metals.

## Materials and methods

### Covalent docking

To model the interaction between 15d-PGJ₂ and the two target cysteine residues (C522 and C423), we employed the covalent docking protocol described in our previous work^24^. Briefly, chemical structure was modified by introducing a covalent bond between the ligand electrophilic carbon and the thiol group of the target cysteine (Fig. 1F). Docking simulations were carried out using the flexible side-chain protocol in AutoDock4^59^, following the standard scripting framework provided with the software. The resulting poses were analyzed using AutoDockTools^60^, and the top-ranked conformation for each condition was selected for further evaluation. A detailed description of the obtained docking conformations is shown in Supplementary Fig. S8.

### Molecular dynamics simulation system preparation

Starting coordinates for sEH were obtained from the Protein Data Bank PDB ID: 6I5E^22^. Initial conformations for the covalently linked 15d-PGJ₂ at residues C522 and C423 were generated via covalent docking. For simplicity, only one monomer of the sEH crystallographic homodimer was used in all computational studies.

In total, three systems were used:

1. **Apo:** apoprotein.
2. **PTG522:** apoprotein with 15d-PGJ₂ covalently bound to C522.
3. **PTG423:** apoprotein with 15d-PGJ₂ covalently bound to C423.

### Replica exchange molecular dynamics (REMD) simulations

Protonation states for all systems were determined using H++^61^, according to a physiological pH of 7. All systems were parametrized using the AMBER ff14SB force field for proteins^62^. 15d-PGJ₂ was parametrized with the General AMBER Force Field (GAFF)^63^, and the partial charges were calculated using the AM1-BCC method^64^. Topologies and parameters were generated using LEaP in AMBER^65^ and converted to GROMACS input files^66^ using ParmEd^67^. The systems were then solvated in a cubic water box of TIP3P water molecules leaving a minimum of 10 Å of water from all sides. Finally, all systems were neutralized with Na^+^ ions.

All systems were minimized using the steepest descend algorithm, and equilibration was conducted in gradual steps, progressively increasing the temperature first under the NVT ensemble and after on the NPT ensemble.

All Replica Exchange Molecular dynamics (REMD) simulations were performed using GROMACS (2021.3)^66^ and standard protocols. Temperature coupling was maintained at desired temperature (299.0–340.13 K) using the velocity rescaling thermostat (v-rescale)^68^ with a time constant of 0.1 ps. Pressure was controlled at 1 bar using the Berendsen barostat^69^ during equilibration with a coupling time of 2.0 ps and an isothermal compressibility of 4.5 × 10⁻⁵ bar⁻¹, and then switched to Parrinello Rahman barostat for production with a coupling time of 1.0 ps and an isothermal compressibility of 4.5 × 10⁻⁵ bar⁻¹. Long-range electrostatic interactions were treated with the Particle Mesh Ewald (PME) method^70^ using a real-space cutoff of 1.0 nm. Bond lengths were constrained using the LINCS algorithm^71^, allowing for an integration time step of 2 fs.

Two independent REMD (Replica Exchange Molecular Dynamics) simulations^72^ were performed for each system. Each REMD run consisted of 25 replicas with temperatures evenly spaced between 299.0 K and 340.13 K. The average exchange rate between adjacent replicas ranged from 25 to 28%. Each simulation was run for 1000 ns, with the PTG423 and PTG522 systems extended to 2000 ns to ensure convergence. Trajectories were reconstructed at the target temperature of 300.63 K.

An additional REMD simulation of 15d-PGJ_2_ bound to the C522 site was performed starting from an alternative docking pose to evaluate pose dependence and ensure that no plausible ligand conformations were overlooked (Supplementary Figs. S8 and S9).

The REMD simulations were performed on the ARCHER2 High-Performance Computer^73^. Starting files and input files for all simulations are provided in the supplementary information.

### Simulation analysis

Simulation analyses were performed using Python libraries MDTraj^74^ and MDAnalysis^75^. Root Mean Square Deviation (RMSD) calculations were carried out using the first frame of each trajectory as the reference structure unless otherwise specified. The Potential of Mean Force (PMF) was calculated to represent the underlying energy landscapes according to equation PMF = −*k*_B_Tln(*P*_i_), where *k*_B_ is the Boltzmann constant^76^, T is the temperature (K), and *P*_i_ is the probability of conformation *i*.

PCA was performed in a supervised manner following K-means clustering and restricted to ligand ring coordinates. The cluster/states delineation analysis of PCA was based on PMF energy values, setting a threshold for the energy barrier separating the states to approximately ~ 1 kcal/mol.

Quantitative analysis of population in different states for C522 was performed by counting the number of frames from the REMD trajectory of each state.

CAVER 3.0^32^ was used to analyze protein tunnels connecting external environment and the catalytic site. The search was initiated from the center of the catalytic site (Y383, Y466, D335) using a probe radius of 0.9 Å. AllohubPy^34^ was employed to detect allosteric signals and identify potential allosteric pathways. AlloHubPy encodes protein conformations of interest using a structural alphabet composed of overlapping four-residue fragments, generating a string representation that preserves local structural changes. Coupling between fragments is then quantified using an information-theoretic approach. Subsequently, fold changes in coupling are evaluated across conditions, with statistical analyses performed over trajectory blocks and replicates to identify differentially coupled residues. Finally, the computed couplings are used to construct a graph, enabling the analysis of allosteric signal propagation. For pathway determination, the most promising residues forming allosteric hubs were selected, and communication pathways connecting these hubs to the vertex (Y383, Y466, D335, D496, H524) were mapped.

Protein-ligand interaction analyses including salt bridge formation, hydrogen bonding, and overall contact mapping were carried out using MDAnalysis^75^. Salt bridges were defined as interactions within 4.5 Å between oxygen atoms of 15d-PGJ_2_ carboxyl group or cyclopentanone ring and the appropriate nitrogen atom on the side chains of lysine or arginine residues. Hydrogen bonds were identified using the HydrogenBondAnalysis^77^ module of MDAnalysis. Overall contact maps were calculated with an inter-atom cut-off of 4.0 Å.

### Protein cloning, expression and purification

Human sEH CTD protein (amino acids 230–555) was cloned as described in previous literature^78^. Mutation was performed using Q5 Site-Directed Mutagenesis Kit (NEB) with primers listed in Supplementary Table S1. The recombinant sEH CTD was expressed in *E.coli* BL21-AI cells using auto-induction medium ZYM5052^79^. A single transformed colony was picked and inoculated in 200 mL of ZYM5052 medium and cultured at 30 °C, 200 rpm overnight. The next day, the pre-culture was diluted to an optical density at 600 nm (OD_600_) of 0.1 in 1L ZYM5052. Cells were cultured at 37 °C, 200 rpm until OD_600_ reached 0.6–1. Protein expression was then induced by adding 0.05% arabinose to the medium and left overnight at 18 °C, 200 rpm. Cells were harvested by centrifugation (5000 g, 30 min, 4 °C) and pellets were kept at −20 °C until further purification.

The sEH CTD proteins were purified as described previously^78^. In brief, the protein was purified sequentially by a nickel-immobilized metal affinity chromatography, a home-made affinity benzylthio-sepharose (BTS) column, and a size exclusion chromatography (SEC). The purified protein was aliquoted and stored in storage buffer (300 mM NaCl, 50 mM Tris-HCl pH 7.4, 1 mM DTT) and kept at -80°C until future usage.

### Covalent complexes preparation

15d-PGJ_2_ was purchased from Cayman (No. 18570) in methyl acetate. The methyl acetate was vacuum evaporated by Concentrator plus (Eppendorf) in V-AQ mode and resuspended in DMSO. The covalent complexes were prepared by mixing 50–300 μg sEH CTD with 15d-PGJ_2_ in a ratio of 1:15 in 0.5–1 mL reaction buffer (25 mM MOPS pH 7.4, 75 mM NaCl). The mixture was left to incubate at 4 °C for 24 h. Covalent 15d-PGJ_2_-sEH complexes were then purified by washing out the excess of unbound 15d-PGJ_2_ in a centrifugal filter unit (Amicon Ultra 0.5 mL, 3000 NMWL) until no ligand can be UV-detected in the flowthrough. Control samples were run side-by-side where sEH CTD was mixed with DMSO.

### Enzymatic specific activity assay

The enzymatic activity of the sEH CTD was measured by a spectrofluorometric method previously described^78^. The assay employs the synthetic substrate 3-phenyl-cyano(6-methoxy-2-naphthalenyl)methylester-2-oxiraneacetic acid (PHOME), which upon sEH enzymatic hydrolysis releases the fluorescent product 6-methoxy-2-naphtaldeyde (6M2N). In brief, the sEH CTD protein stocks were reduced with 15 μM TCEP on ice for 15 min, after which the protein was diluted in 25 mM Tris-HCl (pH 7.4) buffer and added to black 96-well plates to yield final concentrations of 5, 10, 20, 50, 100, and 200 nM. PHOME was added to each well to a final concentration of 10 μM. Fluorescence was monitored using a PolarStar Omega plate reader (BMG Labtech) at 30 °C, with excitation/emission wavelength 330/480 nm, gain 1500, top optic, for 20 cycles of 69-s each. 6M2N accumulation was quantified using a standard curve correlating 6M2N concentration with relative fluorescence units (RFUs). Enzymatic activity was represented in units of (pmol 6M2N)/min/(mg sEH CTD).

### IC50 measurement

The inhibitory effect of metal ions on sEH CTD was measured using the enzymatic activity assay described above. sEH CTD and the mutants thereof were reduced on ice with 10 μM TCEP for 10 min. The reduced proteins were then diluted to a final concentration of 15 nM in 25 mM Tris-HCl (pH 7.4) buffer and transferred to a black 96-well plate. The proteins were incubated with varying concentrations of CuCl_2_ (ranging from 0.1 to 500 μM) for 10 min at room temperature before progressing to fluorescence measurements. The resulting data was analyzed using R and a sigmoidal dose-response model was fitted to determine IC50 values.

### Ultraviolet-visible (UV) and circular dichroism (CD) spectroscopy

The UV absorption and CD spectra were collected using an Applied Photophysics Chirascan Plus spectrometer (Leatherhead, UK). For CD measurements, sEH proteins were analyzed at concentrations ranging from 0.05 to 0.1 mg/mL. Spectra were recorded at 25 °C with a spectral bandwidth of 2 nm, a step size of 1 nm, and an integration time of 1 s per data point. The far-UV CD spectra were smoothed using the Savitzky-Golay method with a smoothing window factor of 4. Protein concentrations were determined from the corresponding UV absorption spectra and used to calibrate the far-UV CD data. Final far-UV CD spectra are reported as mean residue ellipticity, calculated based on the molecular weight and number of amino acid residues of each protein.

### Isothermal titration calorimetry (ITC) experiments

ITC experiments were performed using a MicroCal iTC200 (Malvern Panalytical). The sEH CTD WT and mutants were reduced with 0.25 mM TCEP on ice for 15 min before being dialyzed overnight in ITC buffer (25 mM Tris-HCl pH 7.4, 25 mM KCl) at 4 °C. After dialysis, aggregated protein was removed by centrifugation at 13,000 rpm and 4 °C for 3 min. A CuCl_2_ stock solution of 0.4 M was freshly prepared in ITC buffer. The ITC experiments were run at temperature of 25 °C, stirring speed of 750 rpm, reference power of 5 μcal/s, with a total number of twenty 2-μL injections. In the experiments, 25 μM of human sEH CTD proteins (WT or mutants) were placed in the sample cell and 40 μL of 250 or 500 μM CuCl_2_ were placed in syringe. Control ITC experiments were run by titrating the same amount of CuCl_2_ into ITC buffer. Data were analyzed by MicroCal ITC-ORIGIN Analysis software (Malvern Panalytical) and fit to non-linear curve fitting (NLSF) and one-set-of-sites model. The fitting parameters were Δ*H* (reaction enthalpy change), *K*_D_ (dissociation constant) and *N* (number of binding sites). The reaction entropy was calculated by the software using the relationships Δ*G* = −*RT*·ln*K*_b_ (*R* 1.985 cal mol^−1^ K^−1^, T 298 K) and Δ*G* = Δ*H*-*T*Δ*S*.

### Single-injection kinetic ITC experiments

Single injection method (SIM) kinetic ITC experiments were performed on a MicroCal PEAQ-ITC (Malvern Panalytical) following previous work^35^. The sEH CTD substrate 14(15)-epoxyeicosatrienoic acid (EET) was purchased from Cayman in ethanol. The ethanol was vacuum evaporated using a Concentrator plus (Eppendorf) in V-AQ mode and reconstituted in ITC buffer (25 mM Tris-HCl pH 7.4, 25 mM KCl). The SIM kinetic ITC measurements were run at 25 °C. A single 38-μL injection of 0.5 mM 14(15)-EET was titrated into 250 nM of sEH CTD placed in the sample cell in the presence or absence of 500 nM of CuCl_2_. The injection was completed over 76 s, with a post-injection spacing ranging from 600 to 2000 s. Control experiments were carried out by injecting ITC buffer into protein or 14(15)-EET into ITC buffer. Data were analyzed using the MicroCal PEAQ-ITC Analysis Software (Malvern Panalytical) by fitting to a single injection kinetic model, where kinetic parameters and Michaelis-Mentel plots were obtained.

### Statistics and reproducibility

All experiments in this study, unless specified otherwise, were performed in three independent replicates. All statistical analyses were performed in R. For protein-ligand interaction studies, interaction times (frequencies) were averaged across independent simulation runs, and uncertainty was quantified as the standard deviation (SD) calculated from the two replicates of each simulation system. Data are reported as mean ± SD. For assessing the enzymatic activity of sEH CTD protein, the homogeneity of variance was assured by Levene’s Test and Bartlett’s Test. Statistical significance was computed by one-way ANOVA followed by Tukey’s HSD test. Levels of significance are indicated as following: ns (*p* > 0.05, not significant), * (0.01 < *p* ≤ 0.05), ** (0.001 < *p* ≤ 0.01), *** (0.0001 < *p* ≤ 0.001), **** (*p* < 0.0001).

### Reporting summary

Further information on research design is available in the Nature Portfolio Reporting Summary linked to this article.

## Supplementary information

Supplementary Information

Supplementary data

Description of Additional Supplementary Files

Reporting Summary

42003_2026_10100_MOESM5_ESM

## Data Availability

Starting structures for all MD simulations, along with input files, topologies, and compressed production runs used to generate the data in this paper, are available at Zenodo^80^. Raw values for all figures can be downloaded from the Supplementary Data 1.
