# Supplementary material for: Allosteric ensembles elucidate mechanisms of inhibition in the human soluble epoxide hydrolase
Source: Commun Biol. 2026 May 5;9:931. doi: 10.1038/s42003-026-10100-7 (PMC13350973; doi:10.1038/s42003-026-10100-7)
Supplement: Supplementary file 1 — Supplementary Information [file 42003_2026_10100_MOESM1_ESM.pdf]

Allosteric ensembles elucidate mechanisms of inhibition in the human soluble epoxide hydrolase

Authors

Qiongju Qiu[1]†, Oriol Gracia Carmona[1,2]†, Giancarlo Abis[1], Franca Fraternali[2*] and Maria R Conte[1*]

Affiliations

[1]Randall Centre for Cell and Molecular Biophysics, King's College London, New Hunt's House, Guy's Campus, London SE1 1UL, UK

[2]Institute of Structural and Molecular Biology, University College London, Darwin Building, Gower Street, London, WC1E 6BT, UK.

* Corresponding authors. Emails: sasi.conte@kcl.ac.uk; f.fraternali@ucl.ac.uk

† Equally contributing authors

This PDF file includes:

Supplementary Figure S1 to S9
Supplementary Tables S1

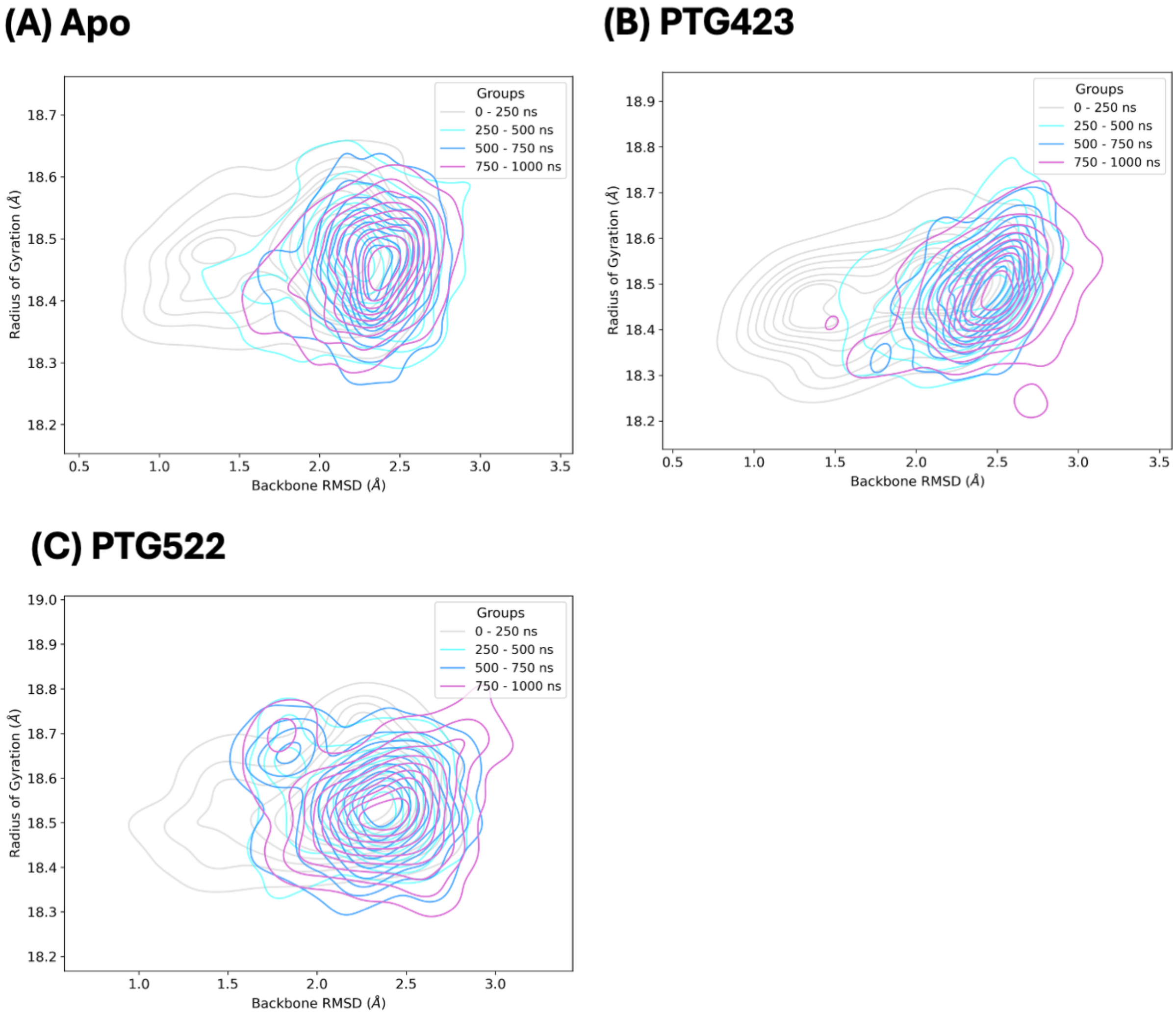

Supplementary Figure S1. REMD simulations reach equilibrium within 1 μs. (A-C) 2D density plots of backbone Root-Mean-Square-Deviation (RMSD) and Radius of gyration (Rg) at increasing cumulative simulation time for system (A) Apo (apoprotein), (B) 15d-PGJ_2 bound to C423 (PTG423) and (C) 15d-PGJ_2 bound to C522 (PTG522). Time intervals (0-250, 250-500, 500-750, and 750-1000 ns) are colored in grey, cyan, blue, and purple, respectively.

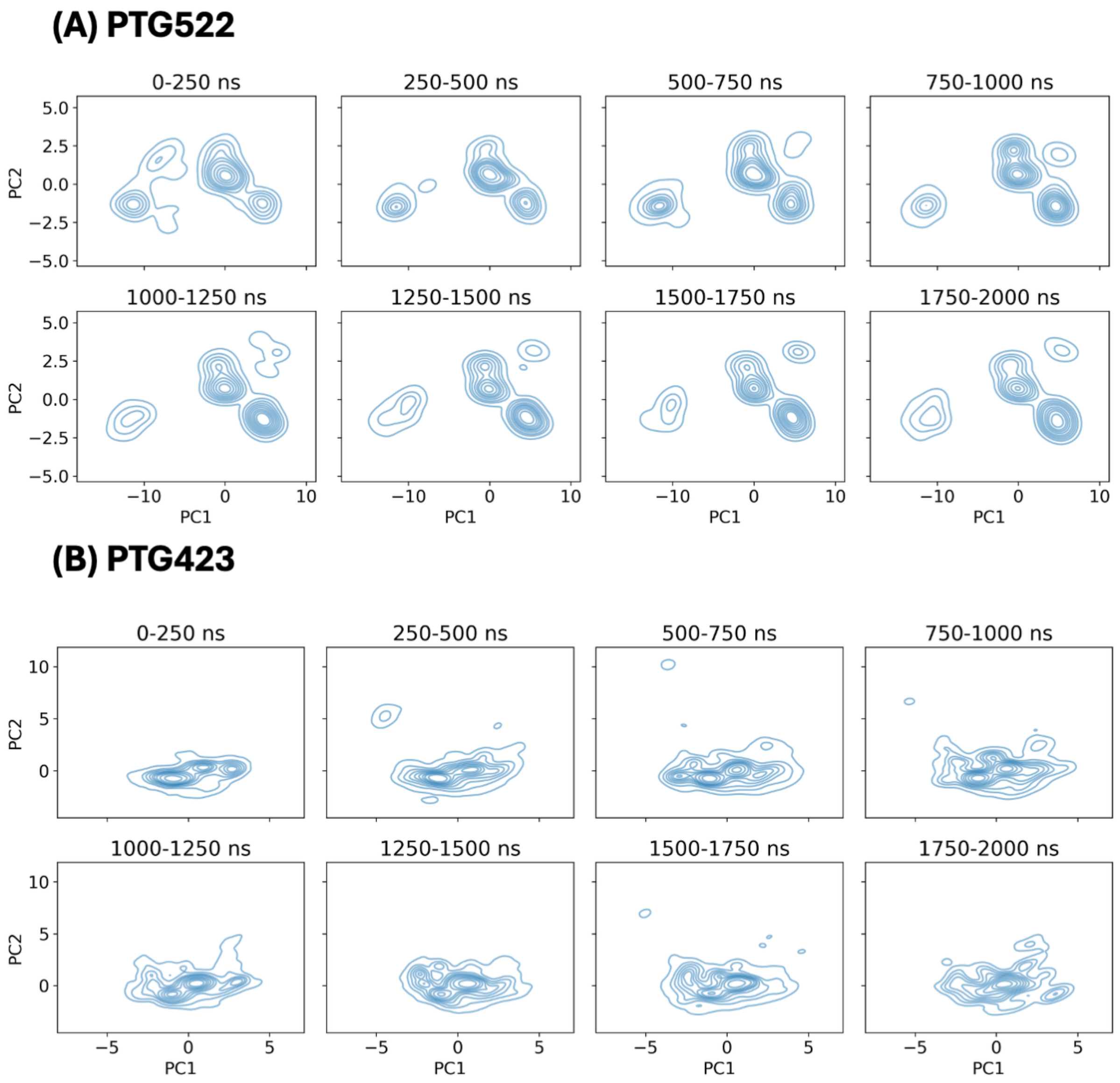

Supplementary Figure S2. Analysis of 2 μs REMD simulations. Extending the REMD simulations to 2 μs confirmed conformational sampling completeness. 2D supervised PCA projections of REMD trajectories based on the center of mass of the 15d-PGJ$_2$ cyclopentanone ring in different cumulative time (0-2 μs) for simulation system (A) PTG522 and (B) PTG423.

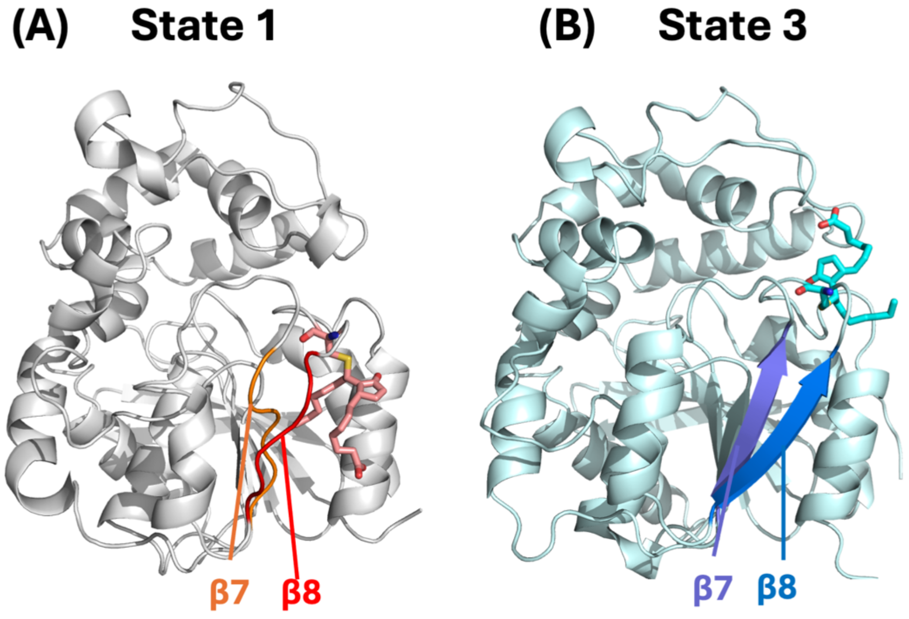

Supplementary Figure S3. Representative snapshots of PTG522 in state 1 and state 3. (A) Representative snapshot of PTG522 in state 1. 15d-PGJ_2 is shown as salmon sticks. β7 and β8 strands are colored orange and red, respectively, while the rest of the protein is shown in white cartoon. (B) Representative snapshot of PTG522 in state 3. 15d-PGJ_2 is shown as cyan sticks. β7 and β8 strands are colored purple and blue, respectively, and the rest of the protein is shown in light cyan.

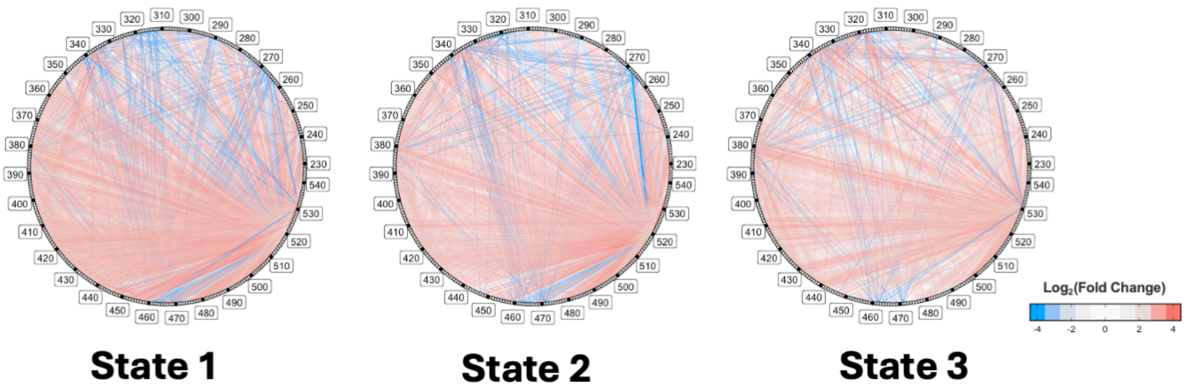

Supplementary Figure S4. The three states of PTG522 showed similar allostery communication with state 3 being the weakest. Log_2 fold-change in signal coupling between each fragment in state 1, 2, and 3 of PTG522 as compared to Apo. Fragments 229–546 are represented as circular dots, labeled every 10 fragments (black dots). An increase or decrease of correlation between two fragments is shown by red or blue lines, respectively.

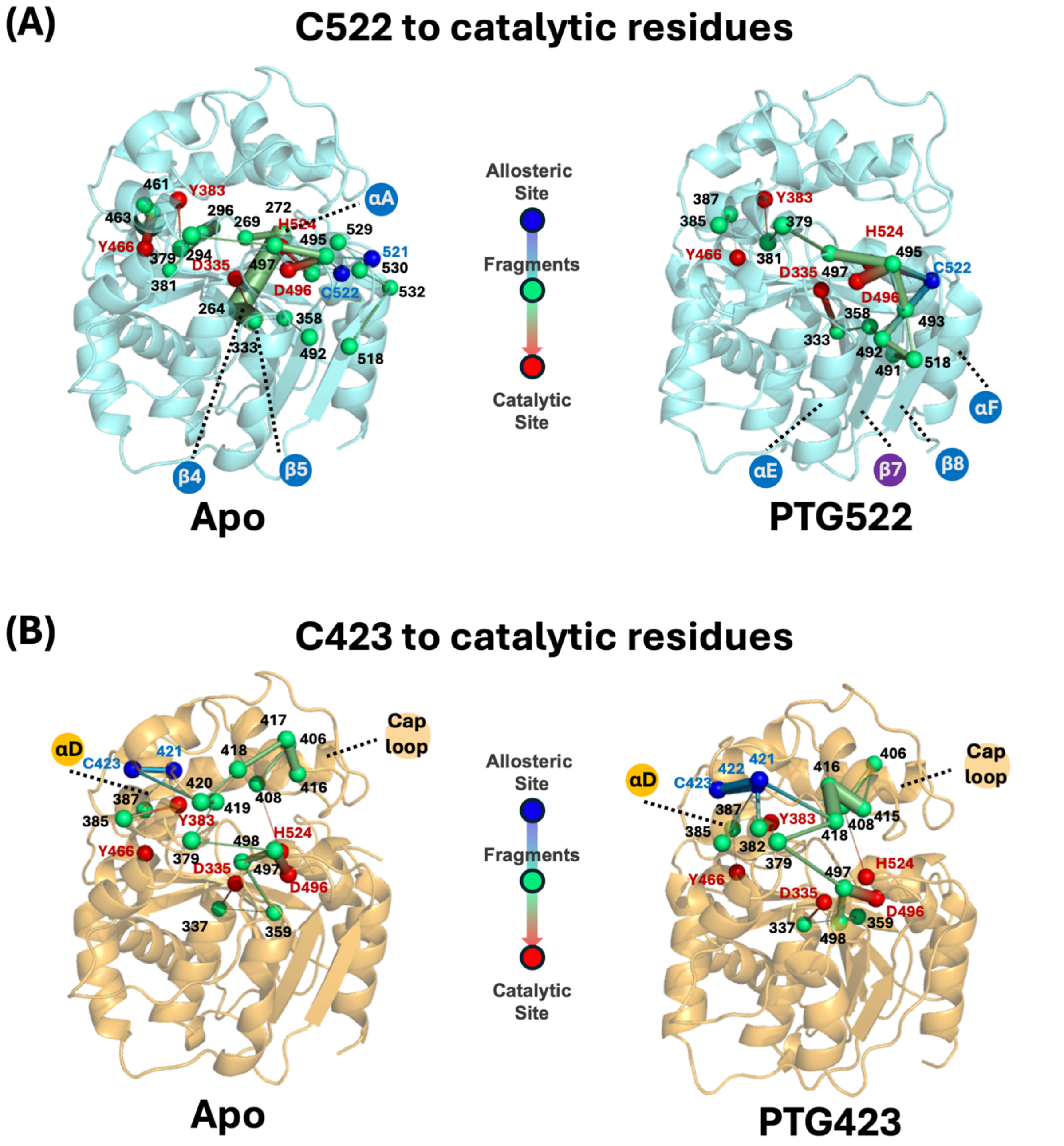

Supplementary Figure S5. Propagation of allosteric signals from the distal sites (C423 or C522) to sEH CTD catalytic site (residues Y383, Y466, D335, D496, H524) in each simulation system (Apo, PTG423, PTG522). (A–B) Shortest pathway of communication from the allosteric site (A) C522 or (B) C423 to the catalytic center of sEH CTD. The allosteric sites, intermediate fragments, and catalytic sites are colored in blue, green, and red, respectively. Thickness of pathway indicates correlation strength. Key protein regions in each system are labelled and highlighted by dashed lines.

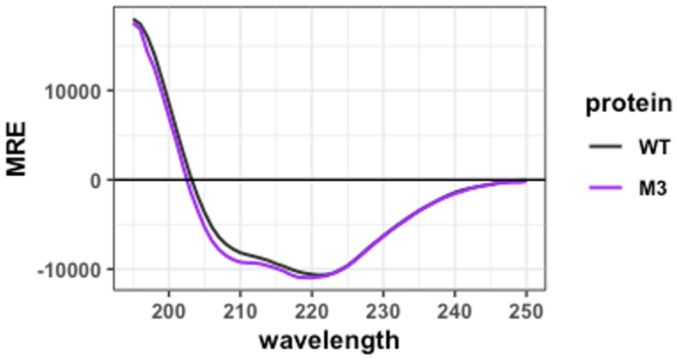

Supplementary Figure S6. Circular dichroism (CD) spectra of sEH CTD WT and mutant M3. Representative far-UV CD spectra were recorded over a wavelength range from 195 to 250 nm. The signal is reported as mean residue ellipticity (MRE). The sEH CTD WT and mutant M3 are shown as black and purple solid lines, respectively. n = 3 independent replicates.

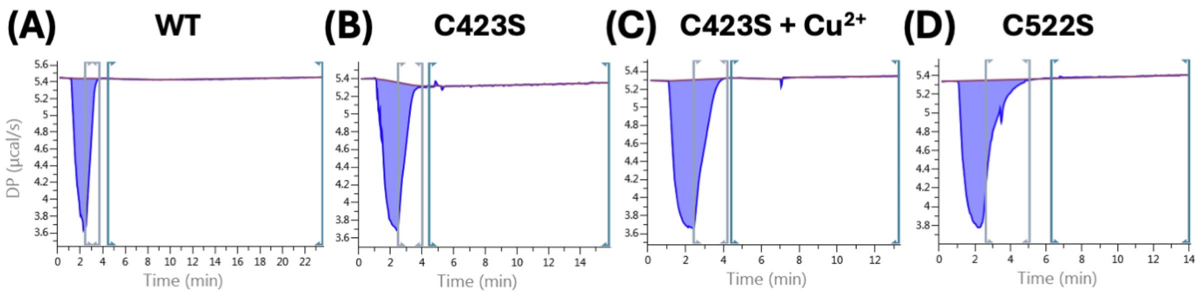

Supplementary Figure S7. Data point selection for Single Injection Method (SIM) Isothermal Titration Calorimetry (ITC) kinetic analysis. (A-D) Representative windows used for fitting of Michaelis–Menten kinetic curves in SIM ITC experiments with 14(15)-EET titration into sEH CTD (A) WT, (B) C423S, (C) C423S (in presence of Cu^{2+}), and (D) C522S. A grey window indicates selected data points for each plot, with the start and the end of the window corresponding to saturated enzyme condition and start of kinetics curve, respectively. Initial injection peaks are excluded from the analysis. n = 3 independent biological replicates.

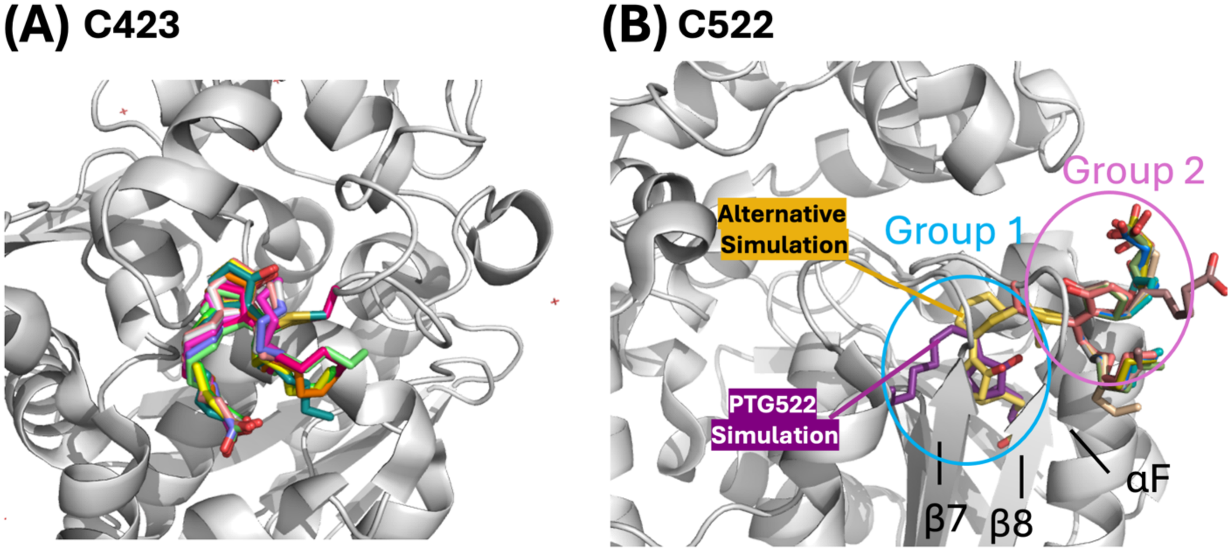

Supplementary Figure S8. Docking poses of 15d-PGJ$_2$ covalently bound to C423 and C522 of sEH CTD, as generated by AutoDock4. (A) Docking poses of 15d-PGJ$_2$ covalently bound to C423. Individual poses are shown as stick representations in different colors. sEH CTD protein is depicted as white cartoon. (B) Docking poses of 15d-PGJ$_2$ covalently bound to C522. Poses are shown as colored stick representations. Group 1 includes two poses in which the ligand is positioned near β7 and β8, whereas group 2 comprises poses with the ligand oriented towards the solvent near αF. The pose within group 1 colored in purple was selected as the starting point for the investigations presented in this work, shown in Figure 2. Notably, group 2 poses recapitulate the PTG522 state 2 conformation observed in our MD simulations (Figure 2). Group 1 contains an alternative starting conformation, shown in yellow, which was used for parallel MD simulations, shown in Supplementary Figure S9.

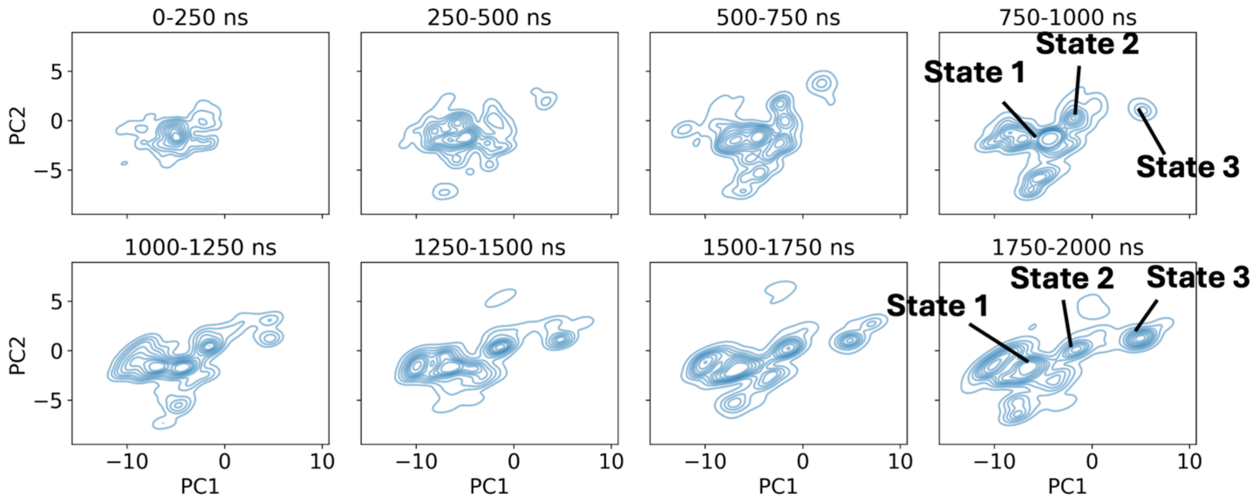

Supplementary Figure S9. Analysis of MD simulations for PTG522 starting from an alternative starting pose. 2D supervised PCA projections with a time interval of 250 ns for the 2000 ns PTGC522 simulation system initiated from an alternative starting conformation for C522, shown in yellow in Supplementary Figure S8. The supervised PCA is projected onto the same coordinate space used for the main PTG522 simulations of this study (Figure 2), identifying three states that correspond to distinct conformational clusters. These are comparable to the three conformational states revealed in the main PTG522 investigations (Figure 2B), although a longer simulation time is required to achieve convergence when starting from the alternative starting pose.

Supplementary Table S1. Forward (F) and Reverse (R) primers for mutagenesis. The mutation sites are highlighted in bold.

Mutation Site	Primer
C423S	(F) CATAAAGTCT**C**TGAAGCGGGAG<br>(R) CATGGATAAAACACTCTCATC
R410A	(F) AAGCCTCTTC**GCG**GCAAGCGATG<br>(R) TTGAAAGTCCGACTCAG
S439A/R440A	(F) CAGCCTC**GCGGCG**ATGGTCACTG<br>(R) TTGAAAGTCCGACTCAG
K495A	(F) CACGGCGGAG**GCG**GACTTCGTGC<br>(R) ACCATCAGGGCCGGA
