# Supplementary material for: Allosteric ensembles elucidate mechanisms of inhibition in the human soluble epoxide hydrolase
Source: Commun Biol. 2026 May 5;9:931. doi: 10.1038/s42003-026-10100-7 (PMC13350973; doi:10.1038/s42003-026-10100-7)
Supplement: Supplementary file 3 — Description of Additional Supplementary Files [file 42003_2026_10100_MOESM3_ESM.docx]

**Description of Additional Supplementary File**

File name: Supplementary data
Description: Supplementary data with all data source for figures and graphs
